# Molecular profiling of the single-cell proteome via gel electrophoresis and 3D single-molecule imaging

**DOI:** 10.1038/s41467-026-74840-0

**Published:** 2026-07-08

**Authors:** Latiefa Kamarulzaman, Sooyeon Kim, Takuya Hidaka, Misaki Tsuchida, Yuichi Taniguchi

**Affiliations:** 1 Graduate School of Pharmaceutical Sciences, The University of Tokyo, Tokyo, Japan; 2 Institute for Integrated Cell-Material Sciences (iCeMS), Kyoto University, Yoshida-Honmachi, Kyoto, Japan; 3 Graduate School of Frontier Biosciences, The University of Osaka, Osaka, Japan; 4 Laboratory for Cell Systems Control, RIKEN Center for Biosystems Dynamics, Osaka, Japan; 5 Current affiliation: Institute of Molecular Biotechnology of the Austrian Academy of Sciences (IMBA), Vienna BioCenter (VBC), Vienna, Austria; 6 Department of Applied Life Sciences, Faculty of Agriculture, Kyoto University, Kitashirakawa Oiwake-cho, Kyoto, Japan; 7 Graduate School of Biostudies, Kyoto University, Yoshida-Honmachi, Kyoto, Japan

**Keywords:** Biotechnology, Proteomic analysis, Proteomics

## Abstract

Recent advances in shotgun proteomics and immunoassays have yielded powerful single-cell proteomics technologies. However, current methods lack the sensitivity required to comprehensively quantify protein abundances in individual cells. Here, we present single-cell PAGE-PISA, an ultra-sensitive proteome profiling strategy that combines gel electrophoresis with 3D single-molecule fluorescence imaging. Our approach labels all proteins in single cells with fluorescent dyes, separates them by electrophoresis, and counts with single-molecule resolution. This technique quantified over 10^7^ protein copies from a single mammalian cell with the sensitivity to detect low-abundance proteins down to 10^5^ copies per species. Single-cell PAGE-PISA successfully classified cells into distinct cell types based on their proteomic profiles. Furthermore, our single-cell proteome data strongly correlated with predicted developmental states during cardiomyocyte differentiation, providing complementary information to single-cell transcriptome data. Together, single-cell PAGE-PISA enables highly sensitive and quantitative proteome profiling at the single-cell level, capturing subtle proteomic differences that distinguish diverse cellular states.

## Abstract (web summary)

Quantifying protein heterogeneity across single cells remains challenging. Here, the authors develop single-cell PAGE-PISA, integrating gel electrophoresis with 3D single-molecule imaging for sensitive and quantitative single-cell proteome profiling to classify cell types and developmental states.

## Introduction

Heterogeneity occurs at multiple levels of molecular biology and is inherent in many biological processes. For many years, the central dogma of molecular biology has been explored primarily through conventional bulk analyses. However, such approaches provide an average measurement (e.g., protein abundance, gene expression) across a population of cells. While bulk analyses have been useful for distinguishing between diseased and healthy tissues^1^, they often reflect dominant biological traits. To address this issue, single-cell technologies have been developed to capture the unique molecular profiles of individual cells and provide insights into cellular heterogeneity. One widely adopted approach is single-cell transcriptome profiling, which captures RNA expression levels across a broad range of transcripts at the single-cell resolution, enabling clustering of diverse cell types and states within complex populations^2–4^. Despite its potential, RNA expression levels cannot accurately predict protein abundance^5–9^. This is because protein abundance is highly dynamic and greatly influenced by various processes, such as protein degradation and post-transcriptional and translational modifications^10,11^. Therefore, it is imperative to perform direct proteome profiling at the single-cell level to comprehensively capture the protein abundance and modifications that are not reflected by RNA expression.

Currently, there are two major approaches to quantifying protein expression levels in single cells, which are mass spectrometry (MS) and antibody-based analysis^12^. So far, MS-based analysis serves as the gold standard for proteomic studies, attributable to its ability to identify, characterize, and quantify proteins with high multiplexity^13^. However, MS does not provide a comprehensive proteomic analysis for single cells due to its limitations in sensitivity. This makes accurate quantification of proteins using MS even more difficult for single cells because of its low protein abundance, between 50 and 300 pg in single mammalian cell^14^, and the fact that proteins cannot be amplified like nucleic acids. This limitation was significantly addressed by the development of SCoPE-MS^15^, which enables the quantification of over 1000 protein groups per single cell. Since then, many MS-based single-cell proteomics technologies have been developed to improve the detection sensitivity^16–24^. Yet, these methods predominantly quantified highly abundant proteins (>10^4^ copies per cell), while low-abundant proteins (10^1^‒10^3^ copies per cell) remain challenging to quantify^6^.

Apart from MS-based analysis, other single-cell protein analysis techniques have also been developed based on antibody labeling^25–27^. In 2014, Herr et al. developed single-cell Western, which involves isolating single cells into microwells, lysing them in situ, separating proteins by electrophoresis, and immobilizing them for protein abundance analysis of target proteins using antibodies. This approach offers high-throughput analysis by enabling simultaneous assay of 1000‒2000 single cells in less than 4 h. This represents an improvement in both sample throughput and measurement time compared to most MS-based single-cell proteomics approaches, albeit sensitivity remains relatively the same. Recent modifications of single-cell Western using nitrocellulose blotting and enzyme-antibody conjugates have lowered the detection limit to approximately 10^3^ molecules per protein species^28^. Despite this progress, its sensitivity is still insufficient for analyzing low-abundance proteins, which remains a key impediment of single-cell Western.

To overcome these limitations, single-molecule fluorescence microscopy offers precise detection and quantification of target molecules. Recently, our group developed a custom-built light-sheet microscope called planar illumination microscope for single-molecule imaging for all purpose (PISA)^29^, which enables 3D single-molecule imaging of all target molecules within a sub-millimeter sample depth. By placing the sample plane above the optical systems for light-sheet imaging and the two objective lenses for illumination and detection below the coverslip at a tilted angle, PISA facilitates counting the number of molecules in the entire volume of a mm-sized biological specimen. In particular, we have shown that PISA can image sub-mm-thick gel at the single-molecule level, achieving attomolar sensitivity. This remarkable sensitivity opens up the possibility for the analysis of single-cell lysates using polyacrylamide gel electrophoresis (PAGE), as even trace amounts of protein from individual cells can be detected.

Here, we present single-cell PAGE-PISA, a highly sensitive strategy for single-cell proteome profiling that integrates PAGE with 3D single-molecule fluorescence imaging using PISA. In this strategy, individual cells are isolated, and proteins are fluorescently labeled, separated by PAGE, and quantified at the single-molecule level. For labeling, we employed *N*-hydroxysuccinimide (NHS)-ester dyes that react with the primary amines of proteins. As the labeling does not rely on antibody, it enables unbiased profiling analysis of the overall cellular proteome without predefined targets, although it does not provide direct identification of the proteins represented in each band. This generates highly sensitive and quantitative electrophoretic band patterns that reflect global protein abundances in individual cells, enabling downstream analyses such as clustering, trajectory inference, and identification of functional cell states solely based on proteome-level variation. To demonstrate the potential of our development, we applied single-cell PAGE-PISA on standard cell lines, PC-3 and U2OS, and explored the temporal changes of cellular proteomes during cardiomyocyte differentiation from human induced pluripotent stem cells (hiPSCs) by tracking their progression through different developmental stages. The resulting proteome profile revealed gradual proteomic changes across single cells that aligned with the progression from pluripotency to differentiated states. Unsupervised clustering and pseudotime analysis further uncovered subpopulations along the differentiation trajectory, which is often difficult to resolve using transcriptomic analysis or marker-based approaches.

## Results

### Establishment of the single-cell PAGE-PISA workflow

**Fig. 1:**
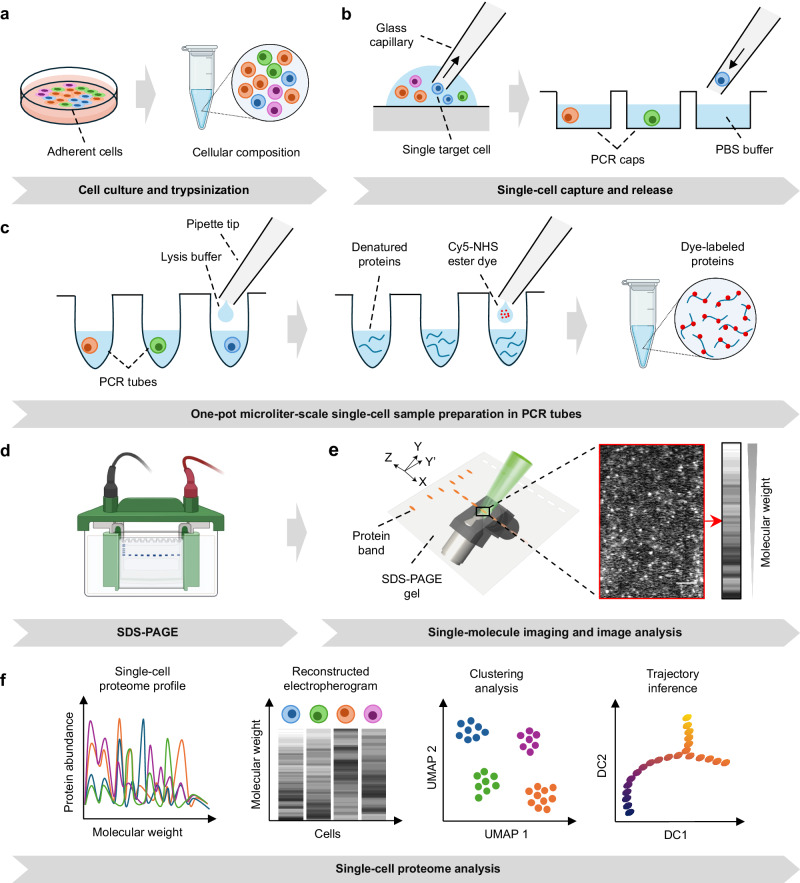
Workflow of single-cell PAGE-PISA. **a** Firstly, cells are dissociated, washed three times, and suspended in PBS at a concentration appropriate for single-cell collection. **b** Then, the single cells are manually isolated from a droplet of cell suspension using an inverted microscope equipped with a cell picker (TOPick 1-cell handling system) and transferred into PCR caps containing PBS. The isolated single cells are spun down to ensure they settle at the bottom of the PCR tubes. **c** After cell lysis, the proteins are labeled with Cy5-NHS ester dye solution at 15 °C for 45 min. To inhibit the reactivity of excess dye, quencher solution is added to the protein sample, followed by a second incubation at 15 °C for 45 min. All steps are performed on ice. The molar ratio of the dye to quencher solution used in the single-cell sample preparation is 1:10. **d** The dye-labeled proteins from each single cell are loaded into the well and separated by molecular size using SDS-PAGE. **e** After protein separation, the dye-labeled proteins in the polyacrylamide gel are visualized using PISA with a 647 nm excitation wavelength. Each detected single molecule observed in the PISA image represents a single dye-labeled protein. Fluctuations in the number of single molecules are observed along the migration path, which correlate with protein bands of different abundances. Subsequent image analysis, including background subtraction, denoising filter, and spot detection, enables the reconstruction of a detailed proteome profile for each single cell. Scale bar: 50 µm **f** Schematic diagrams depicting different single-cell proteome analyses that can be achieved with single-cell PAGE-PISA. Figure 1a, c, d, were created in BioRender. Kamarulzaman, L. (2026) https://BioRender.com/m566srs. In Fig. 1e, the microscope design was created using Rhinoceros®.

To provide a streamlined proteomic strategy for single cells, we established a complete workflow of single-cell PAGE-PISA: (1) cell preparation, (2) manual isolation of single target cells, (3) one-pot sample preparation in PCR tubes, including cell lysis and protein labeling with dye, (4) SDS-PAGE for protein separation, and (5) volumetric single-molecule imaging of dye-labeled proteins in polyacrylamide gel using PISA (Fig. 1).

In the workflow, cells are first dissociated from the culture dish, washed several times, and suspended in phosphate-buffered saline (PBS) solution (Fig. 1a). Then, a droplet of cell suspension is deposited onto a slide glass, from which the single cells are manually isolated using an inverted microscope equipped with a TOPick 1-cell handling system (i.e., low-binding coated micro-glass needle with a tip diameter of 30 µm and aspirating volume of 100 pL) (Fig. 1b and Supplementary Fig. 1a). The isolated single cells are dispensed into PCR caps containing a droplet of PBS and spun down to ensure the droplet settles at the bottom of the tube. With manual single-cell isolation, we can precisely control cell occupancy by eliminating the possibility of droplets containing multiple or no cells. The entire process of identifying, picking up, and releasing each target cell takes less than 2 min. Furthermore, we perform single-cell sample preparation on ice without direct contact between pipette tips and protein samples (Fig. 1c). Instead, all reagents, including lysis buffer, protease inhibitor, Cy5-NHS ester dye, and quencher solution, are dispensed along the tube wall without touching the samples directly and mixed by spinning down. This “all-in-one” sample preparation method enhances protein recovery for quantitative single-cell proteome profiling by considerably minimizing potential loss due to surface adsorption and sample transfer.

Next, to separate proteins by molecular weight, we use a commercial 20-well polyacrylamide gel designed for low sample volume with a capacity of up to 8 µL per loading well (Fig. 1d, Supplementary Fig. 1b). The use of a standard polyacrylamide gel with an 8 cm length, instead of preparing a miniaturized gel like the single-cell Western system^26^, improves the separation resolution of the band profiles by resolving proteins with close molecular weights. Meanwhile, the narrow loading well of approximately 4 mm width further concentrates the protein samples within each band along the migration path. As protein bands from single cells cannot be observed by the standard gel imager, we optimized the electrophoresis conditions for single cells (e.g., time, voltage, and current) using the bulk cell lysates in a preliminary experiment. The resulting band profiles from bulk cell lysates, visualized by the standard gel imager allowed us to determine the exact migration pattern and position of each protein band on the polyacrylamide gel (Supplementary Fig. 2a). After single-cell electrophoresis, the gel is carefully cut, transferred onto a film substrate, and placed on a microscopic stage (Supplementary Fig. 1c, d).

Finally, we perform 3D single-molecule imaging using PISA to detect and quantify the dye-labeled proteins, which can be observed as clear diffraction-limited spots during imaging (Fig. 1e). We focus on the middle molecular weight region, approximately from 20‒40 to 100‒150 kDa, which corresponds to a 5 cm gel length (Supplementary Fig. 1d). The region of interest is determined by the size of the metal stage and the balance between measurement time and sufficient proteome information. The single-molecule imaging is conducted with a scan speed of 80 µm/s, resulting in approximately 10 min of imaging time per single cell. Fluctuations in the number of dye-labeled proteins are observed along the migration path during imaging, which reflects protein bands with differing protein abundances across molecular weights (Supplementary Fig. 2c and Supplementary Movie 1). Detecting and quantifying the dye-labeled proteins at the single-cell level allows us to gain insights on protein expression profiles, cell type classification, and pseudotime analysis (Fig. 1f).

To evaluate the sensitivity and quantitative accuracy of the PISA measurement, we imaged and quantified the number of dye-labeled proteins from highly diluted bulk HeLa and U2OS cell lysates, equivalent to a single-cell concentration. Note that the bulk cell lysates were diluted to single-cell level based on the estimated initial number of the cell suspension. We found that the reconstructed electropherogram obtained by single-molecule imaging was in good agreement with the SDS-PAGE band profile visualized by the standard gel imager (Supplementary Fig. 2b and Supplementary Fig. 2a, respectively). Quantitative analysis revealed that the adjusted protein counts across molecular weights were consistent in three technical replicates, with a median coefficient of variation (CV) of 6.3% and 7.8% for HeLa and U2OS, respectively (Supplementary Fig. 2d, e). The analytical reproducibility was assessed by computing the Pearson correlation of the adjusted protein counts across molecular weights between replicates, which yielded coefficients larger than 0.96 (Supplementary Fig. 2f). To assess the linearity of the method, we performed an experiment using highly diluted bulk cell lysates equivalent to 1, 2, 4, and 8 cells. Bulk cell lysates were used to minimize the variability from cell-to-cell differences in protein content or cell cycle stages, thus ensuring controlled and consistent protein input. Our result demonstrated linearity (*R*² = 0.998) between cell equivalents and total adjusted protein counts (Supplementary Fig. 2g, h). The ability to maintain linearity down to single-cell equivalent shows robust quantitative accuracy and great detection sensitivity of our system, even in highly diluted samples.

Lastly, to assess the detection sensitivity of single-cell PAGE-PISA, particularly for low-abundance proteins, we conducted a lysate spike-in experiment using transferrin (77 kDa) as a model protein and evaluated its detectability in a complex cell lysate background (Supplementary Fig. 3a). We successfully detected as little as 50 fg of transferrin, corresponding to approximately 1.53 × 10^5^ protein molecules (Supplementary Fig. 3b). These results demonstrate that single-cell PAGE-PISA can reliably detect and quantify proteins present at copy numbers in the 10^5^–10^6^ range within a complex cell lysate environment.

### Single-cell proteome profiling of mammalian cells

**Fig. 2:**
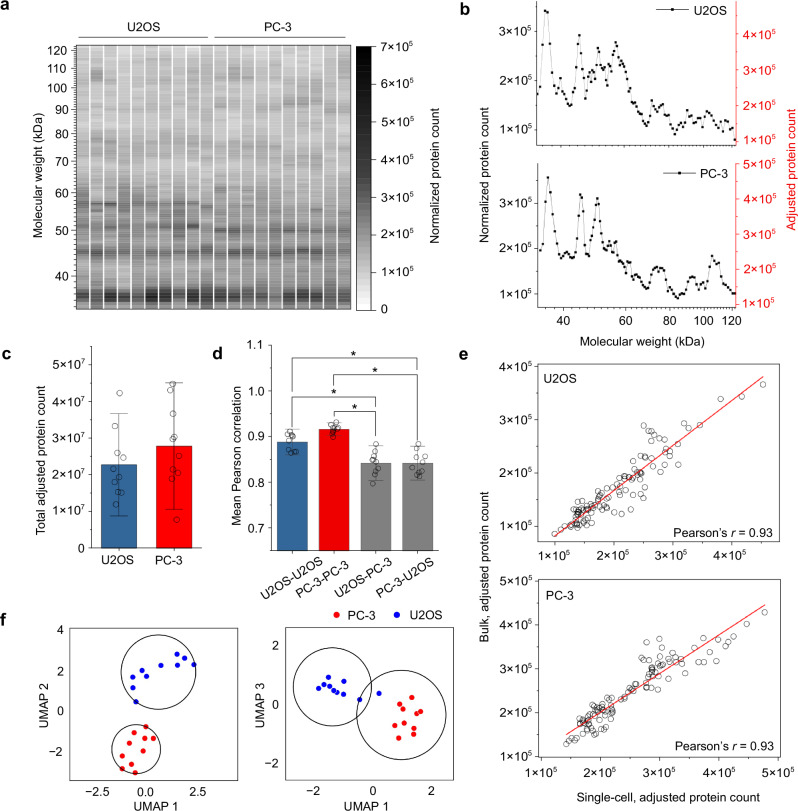
Single-cell PAGE-PISA of single U2OS and PC-3 cells. **a** Reconstructed single-cell electropherogram of U2OS and PC-3 cells obtained by single-cell PAGE-PISA. The electropherogram was reconstructed based on the normalized protein counts from 34 to 122 kDa. **b** Representative proteome profile of single U2OS and PC-3 cells. The *y*-axes represent the normalized (left) and adjusted (right) protein counts from a single cell across molecular weights, respectively. **c** Total adjusted protein count for each cell type. **d** Mean Pearson correlation of adjusted protein counts across molecular weights between single cells, within and across cell types. In (**c,** **d**), error bars represent ± the standard deviation of the mean. **e** Comparisons of the mean adjusted protein count between single cells and technical replicates of highly diluted bulk cell lysates equivalent to single-cell level. The red lines represent the line of best fit, showing a positive correlation between single-cell and bulk measurement. **f** Two-dimensional UMAP projections based on electrophoretic protein band profiles from individual U2OS (blue) and PC-3 (red) cells, shown as UMAP 1 vs. UMAP 2 and UMAP 1 vs. UMAP 3. Clusters were identified using *k*-means. Number of biological replicates (single cells): *n* = 10 for U2OS and *n* = 10 for PC-3, number of technical replicates (bulk cell lysates): *n* = 3 for U2OS and *n* = 3 for PC-3 cells. Statistical significance was determined by the one-way Welch’s ANOVA followed by the Games-Howell post-hoc test. *p ≤ 0.05. Source data are provided as a Source data file.

Next, we performed single-cell proteomic analysis using single-cell PAGE-PISA on two different tumor cell lines, U2OS and PC-3 (Fig. 2). The single cells were manually isolated, prepared in PCR tubes, and the dye-labeled proteins were separated by SDS-PAGE and imaged by PISA. The imaging was performed at the middle molecular weight region, covering approximately 40‒50% of the cellular proteome. The electropherogram was reconstructed from the PISA images to provide a comprehensive view of the protein expression profiles across multiple single cells (10 biological replicates for each cell type) (Fig. 2a). In practice, our single-cell PAGE-PISA could quantify dye-labeled proteins from a 2 mm^3^ gel volume (0.2 × 0.2 × 50.0 mm^3^). This translates to 10^3^‒10^4^ dye-labeled proteins per band, corresponding to approximately 2 fg, and a total of 10^5^ dye-labeled proteins per single cell. Ideally, imaging the entire migration path (4.0 × 1.0 × 50.0 mm^3^) could theoretically increase these numbers to 10^5^‒10^6^ dye-labeled proteins per band, which corresponds to approximately 200 fg, and a total of 10^7^ dye-labeled proteins in a single mammalian cell (Fig. 2b, c and Supplementary Fig. 4a). This detection sensitivity far surpasses that of conventional staining methods for SDS-PAGE like Coomassie Brilliant Blue (CBB) and silver staining, with detection thresholds of 8‒10 ng^30^ and 0.1‒0.5 ng^31–33^ per protein band, respectively. Thus, these methods were insufficient to visualize protein bands from single-cell samples, unlike single-cell PAGE-PISA which can detect protein bands with atto-gram amounts^29^. Thereby, the detection sensitivity was improved by 3‒5 orders of magnitude over silver staining and CBB, enabling the detection of even 1% of the total protein abundance.

Compared to the estimated 6 × 10^9^ protein copies per HeLa cell^34^, the total protein copies quantified by single-cell PAGE-PISA fall short by two orders. The difference between practical and theoretical values may stem from technical limitations during quantification. For instance, residual dyes that are not completely quenched may weakly interact with the polyacrylamide gel during electrophoresis, causing a slight increase in the overall background signal (Supplementary Fig. 5a, b and Supplementary Movie 2). As a result, some dye-labeled protein molecules that are not distinctive from the background may fall below the detection threshold and fail to be quantified (Supplementary Fig. 5c). Furthermore, if two dye-labeled protein molecules are within the optical diffraction limit (i.e., a few hundred nanometers), especially in highly dense protein bands, they cannot be resolved and will be detected as a single molecule due to the diffraction limit. Nevertheless, the strong mean Pearson correlation of adjusted protein counts across molecular weights between single cells from the same cell types (*r* > 0.89) demonstrates great reproducibility and quantitative accuracy of single-cell PAGE-PISA (Fig. 2d and Supplementary Fig. 4b).

Next, we conducted a detailed peak fitting analysis to determine the peak capacity and mass resolution of single-cell PAGE-PISA. We identified 21 distinct Gaussian peaks between 34 and 127 kDa in a representative single-cell proteome profile (Supplementary Fig. 6a). For proteins in the 34–60 kDa range (peaks #1–8), the average mass resolution was 1.34 kDa (FWHM) (Supplementary Fig. 6b). For proteins in the 60–127 kDa range (peaks #9–21), the resolution declined to an average of 2.69 kDa, likely due to reduced gel migration efficiency for large proteins.

We further assessed the accuracy and reliability of our single-cell measurement by comparing the single-cell protein expression profile to those of highly diluted bulk cell lysates equivalent to a single-cell concentration from the same cell types. We averaged the adjusted protein counts across multiple single cells (*n* = 10) and technical replicates of highly diluted bulk cell lysates (*n* = 3), then compared their mean adjusted protein count across molecular weights (Supplementary Fig. 4c). We observed a strong Pearson correlation of mean adjusted protein counts between single cells and highly diluted bulk cell lysates for both U2OS and PC-3 cells (*r* > 0.93) (Fig. 2e). The observed correlation showed that the single-cell protein quantification by single-cell PAGE-PISA was in good agreement with that from bulk cell lysates diluted to the single-cell level, indicating the robustness and reliability of our single-cell measurement.

Lastly, we visualized the 2D projections of single U2OS and PC-3 cells, specifically in UMAP 1 vs. UMAP 2 and UMAP 1 vs. UMAP 3 plots (Fig. 2f). Our results showed clear separation between the two cell lines, indicating that the clustering captured meaningful biological differences and was not confined to higher-order dimensions. This demonstrates that single-cell PAGE-PISA can effectively identify and classify different cell types solely based on their proteome.

### Single-cell proteome profiling of cardiomyocyte differentiation from human induced pluripotent stem cells

The ability of hiPSCs to self-renew and differentiate into various cell types and tissues has garnered significant interest, especially in the pursuit of heart regenerative therapies^35,36^. Numerous studies have shown that protein expressions of hiPSC-derived cardiomyocytes (hiPSC-CMs) exhibit dynamic fluctuations and temporal changes during differentiation and maturation^37–39^.

**Fig. 3:**
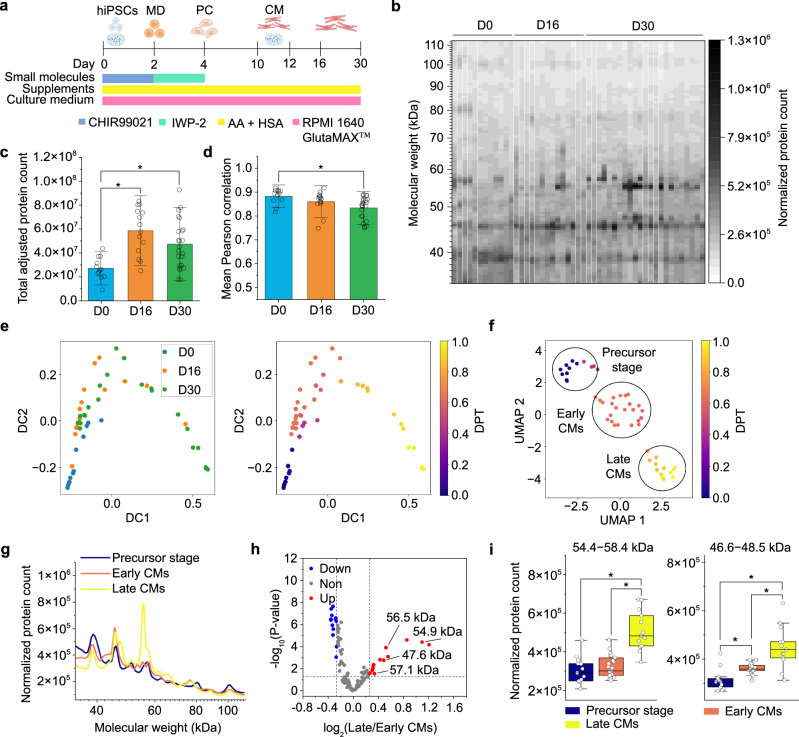
Application of single-cell PAGE-PISA to cardiomyocyte differentiation from human induced pluripotent stem cells. **a** Schematic protocol of cardiomyocytes transitioning from pluripotent state towards cardiac lineage. hiPSCs human induced pluripotent stem cells, MD mesoderm, PC progenitor cells, CM cardiomyocytes, AA ascorbic acid, HSA human serum albumin. The culture dish represents the day when hiPSC-CMs were transferred to a freshly coated plate. **b** Single-cell electropherogram of hiPSCs (D0) and hiPSC-CMs (D16 and D30) were reconstructed based on the normalized protein counts from 34 to 112 kDa. **c** Total adjusted protein count for each sampling day. **d** Mean Pearson correlation of adjusted protein counts across molecular weights between single cells, within and across sampling days. In (**c,** **d**), error bars represent ± the standard deviation of the mean. Number of biological replicates: *n* = 12 for D0, *n* = 14 for D16, and *n* = 23 for D30. **e** Diffusion maps showing the developmental trajectory from pluripotency to differentiated states. The single cells were colored according to their sampling day (left) and DPT (right). The trajectory starts from the root cell (blue) that is located at the left terminal and progresses towards the most differentiated cell (yellow). DC and DPT refer to diffusion components and diffusion pseudotime, respectively. **f** UMAP analysis with *k*-means clustering of the DPT-assigned single cells. Three clusters were identified, representing different developmental stages, precursor stage (*n* = 13), early cardiomyocytes (*n* = 23) and late cardiomyocytes (*n* = 13). **g** Average single-cell proteome profile for each developmental stage. **h** Volcano plot showing upregulated and downregulated protein bands in late cardiomyocytes compared to early cardiomyocytes. Differential protein bands were determined by Welch’s two-sided, two-sample *t*-test (*p* < 0.05, fold change >1.2). **i** Comparisons of the quantitative expression levels of two protein bands, 54.4‒58.4 kDa and 46.6‒48.5 kDa, across three developmental stages. The box plots show median values (central line), interquartile range (box edges), and whiskers extending up to 1.5 times the interquartile range. Individual data points are overlaid. In (**g**–**i**), single cells from the clusters defined in (**f**) were analyzed (*n* = 49 cells). Statistical significance was determined by the one-way Welch’s ANOVA followed by the Games-Howell post-hoc test. *p ≤ 0.05. Source data are provided as a Source data file. Figure 3a was created in BioRender. Kim, S. (2026) https://BioRender.com/ejy1q5r.

To highlight the potential applicability and versatility of single-cell PAGE-PISA in detecting global proteome changes during dynamic processes, we performed single-cell proteome profiling analysis on hiPSCs as they progressed from pluripotency through stage-specific transitions during cardiomyocyte differentiation (Fig. 3a). We observed cardiac contraction in hiPSC-CMs as early as day 7, indicating the successful differentiation process. To improve cardiomyocyte purity in culture, the hiPSC-CMs were carefully dissociated and transferred to a freshly coated plate between days 10 and 12. Cells were harvested on different days, including day 0 (hiPSCs), 16, and 30 (hiPSC-CMs), referred to as D0, D16, and D30, respectively. In total, 49 single cells were collected, prepared, and analyzed with single-cell PAGE-PISA (*n* = 12, 14, and 23 for D0, D16, and D30, respectively) (Fig. 3b). Quantitative analysis of single hiPSC-CMs from D16 and D30 revealed 1.7‒2.2 times higher total protein abundance than D0, likely due to the increased cell size and complexity in specialized cells (Fig. 3c). In addition, we observed a strong mean Pearson correlation of adjusted protein counts across molecular weights among D0 single cells (*r* = 0.88), implying a consistent single-cell protein expression levels in the undifferentiated state (Fig. 3d). Meanwhile, the mean correlation of adjusted protein counts decreased by approximately 2.6% and 5.5% in D16 and D30 single cells, respectively, suggesting stochastic occurrences in individual cells during cardiomyocyte differentiation. Using all the normalized protein counts from 34 to 112 kDa, principal component analysis (PCA) separated the cells in a time-wise manner along PC1, which accounts for 47.2% of the total variance (Supplementary Fig. 7a). Notably, the data points from different batches did not form distinct clusters in the PCA plot, indicating that batch effects were negligible and did not confound the temporal separation.

We next sought to investigate the cell progression from pluripotency towards differentiated states by pseudotemporal ordering of the dynamic cells across a developmental lineage^40^. Briefly, cells were presented on a diffusion map based on the cell-to-cell transition probabilities, positioning those with higher transition probabilities closer to each other (Fig. 3e, left). The trajectory was inferred by computing diffusion pseudotime (DPT) for each cell relative to the root cell, often considered the starting point of the differentiation process, and cells were ordered along the pseudotemporal trajectory to track their developmental progress during cardiomyocyte differentiation (Fig. 3e, right). As anticipated, the D0 single cells were in close proximity to one another on the diffusion map, indicating homogeneous protein expression profiles among the single cells. In contrast, cells from D16 and D30 appeared more spread out across the developmental trajectory, with D16 and D30 primarily dominating the early and terminal end of the trajectory, respectively. This reflects the temporal dynamics of proteome changes as cells differentiate from early to later stages. We performed UMAP analysis on DPT-assigned single cells and identified three clusters (Fig. 3f and Supplementary Fig. 7b). Precursor stage (*n* = 13) consisted predominantly of D0 cells (*n* = 12, 92.3%), while early (*n* = 23) and late cardiomyocytes (*n* = 13) were mainly composed of D16 and D30 cells (*n* = 10 and *n* = 10, 43.5% and 76.9%, respectively).

We further visualized the single-cell proteome profiles across different developmental stages to identify differentially regulated protein bands (Fig. 3g, Supplementary Fig. 8a). By comparing the protein expression differences between early and late cardiomyocytes, we identified two protein bands that were differentially regulated in late cardiomyocytes (Fig. 3h, Welch’s two-sided, two-sample *t*-test, *p* < 0.05, fold change >1.2). Notably, the protein bands at 54.4‒58.4 kDa and 46.6‒48.5 kDa were significantly expressed in late cardiomyocytes compared to the precursor stage and early cardiomyocytes (Fig. 3i). To infer the molecular identity and interpret changes of the observed protein bands, we integrated our single-cell PAGE-PISA data with complementary single-cell omics and immunofluorescence measurements. We first evaluated candidate proteins potentially contributing to the 54.4–58.4 kDa band detected at D30 by cross-referencing our single-cell PAGE-PISA data with publicly available single-cell transcriptomic and single-cell MS datasets^41,42^. Candidate proteins were filtered based on molecular weight and ranked by abundance in each dataset (top 10 at D30 and D21, respectively) (Supplementary Fig. 8b–d, Welch’s two-sided *t*-test, *p* < 0.05, fold change >1.4). Five candidates were selected for immunofluorescence imaging at D0 and D30 based on these rankings and antibody availability (Supplementary Table 1). Importantly, MT-CO1 and P4HB showed increased protein abundance at D30 relative to D0, consistent with enhanced mitochondrial respiratory activity and prevention of ER stress and protein misfolding, respectively^43,44^ (Supplementary Fig. 9a, b). In contrast, PHGDH showed decreased protein abundance at D30, consistent with reduced PHGDH levels reported in hiPSC-CMs^45^. PDIA3 abundance also decreased at D30, and given its reported association with proliferative activity^46^, this reduction may indicate decreased proliferation in differentiated cardiomyocytes and a transition toward a more post-mitotic state. These results suggest that the protein band observed within 54.4–58.4 kDa at D30 is predominantly contributed by MT-CO1 and P4HB, whereas PHGDH and PDIA3, despite their high abundance in single-cell transcriptomics and single-cell MS datasets, contribute only minimally to the protein band expression. Immunofluorescence imaging further revealed cell-to-cell variability in ATP5B abundance at D30 despite no directional change at the population level, suggesting heterogeneous activation of mitochondrial ATP synthesis at the single-cell level.

### Comparative analysis between single-cell proteome and transcriptome during cardiomyocyte differentiation

**Fig. 4:**
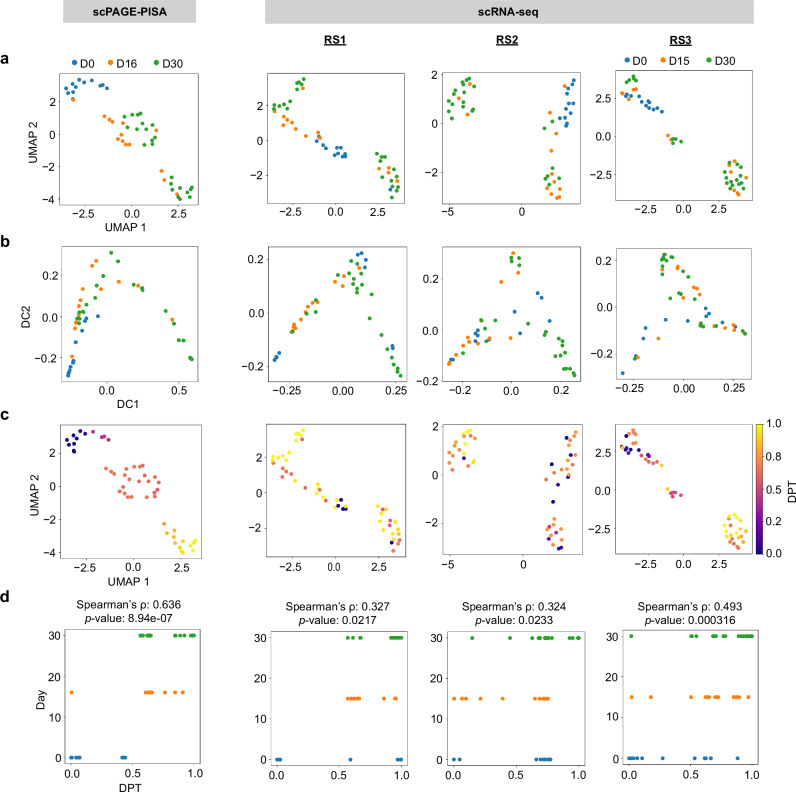
Comparative analysis between single-cell proteome and transcriptome during cardiomyocyte differentiation. **a** UMAP projection of single cells based on the overall similarity of gene or protein expression. **b** Diffusion maps showing single cells arranged along a pseudotemporal trajectory based on the transition probabilities between two cells, positioning those with higher transition probabilities closer to each other. **c** UMAP projection of the same single-cell embedding as in (**a**), with cells colored by their DPT values. **d** Correlation between sampling day and DPT. For scRNA-seq data, three RS were generated by randomly selecting 49 single cells from the entire dataset. In total 10,484 genes were used for the analysis, corresponding to proteins with molecular weights within 37‒126 kDa. DC refers to diffusion components, DPT refers to diffusion pseudotime, and RS refers to random sample. Statistical significance was determined using a two-sided Spearman correlation test. Number of biological replicates for single-cell PAGE-PISA: *n* = 12, 14, and 23 for D0, D16, and D30, respectively. Number of biological replicates for scRNA-seq: *n* = 12, 14, and 23 for D0, D15, and D30, respectively. Source data are provided as a Source data file.

As proteome and transcriptome measurements are not always correlated^5–9^, we next compared the extent to which our single-cell proteome data obtained by single-cell PAGE-PISA are in agreement with reported single-cell RNA sequencing (scRNA-seq)^41^ during cardiomyocyte differentiation. The scRNA-seq data was chosen as the sampling days when the single cells were collected closely matched our single-cell PAGE-PISA. We analyzed both datasets with an equal sample size (*n* = 49) for accurate comparison. Three random samples (RS) were generated from the scRNA-seq data from 37 to 126 kDa, with each RS consisting of 49 randomly selected single cells from three different sampling days (*n* = 12, 14, and 23 for D0, D15, and D30, respectively). UMAP analysis revealed a consistent spatial distribution of the cells at both proteome and transcriptome levels (Fig. 4a). The D0_protein_ and D0_RNA_ populations formed a distinct cluster, with cells in close proximity to each other, implying that both populations exhibit stable developmental states with consistent expression profiles. Meanwhile, D16_protein_, D30_protein_, D15_RNA_, and D30_RNA_ populations displayed two separate clusters that were distinctive from D0 but did not otherwise cluster with respect to their sampling days. These results indicate that although D16_protein_, D30_protein_, D15_RNA_, and D30_RNA_ populations share similar expression profiles on UMAP space, they reflect transitional states or different developmental stages during cardiomyocyte differentiation.

We expect the spatial distribution of these states can be better resolved on a pseudotemporal scale. Hence, we ordered the dynamic cells along a developmental trajectory from pluripotency to differentiated states at both proteome and transcriptome levels (Fig. 4b and Supplementary Fig. 10b, c). As expected, the majority of D30_protein_ and D30_RNA_ single cells were located at the terminal end of the trajectory, distal from the root cell, signifying their progression into a fully differentiated state. While D0_protein_ population clustered closer to the root cell on the diffusion map, we observed greater variations of D0_RNA_ population in all three RS, with cells sparsely distributed across the developmental trajectory. The D0_RNA_ population, however, can be clearly distinguished from D15_RNA_ and D30_RNA_ populations when increasing the sample size from 49 to 3000 single cells (Supplementary Fig. 10a). This emphasizes the need for a larger sample size of scRNA-seq data^47^ to capture the full spectrum of biological variability.

Lastly, we sought to investigate how well the inferred DPT correlates with the sampling day (referred to as real-time) during cardiomyocyte differentiation at both proteome and transcriptome levels. To this end, we visualized the DPT-assigned single cells on UMAP and assessed the correlation between the DPT and real-time (Fig. 4c, d). We observed a strong concordance between DPT_protein_ and real-time_protein_ (Spearman’s *ρ* = 0.64), while relatively lower concordance between DPT_RNA_ and real-time_RNA_ in all three RS (Spearman’s *ρ* < 0.49) (Fig. 4d). These results demonstrate that the directional changes of the developmental states at proteome level are generally consistent whether determined by DPT or real-time and that both recapitulate the expected temporal changes during cardiomyocyte differentiation. While several D15_RNA_ and D30_RNA_ cells displayed a wide dynamic range of DPT values due to the different progression rate during the differentiation process, it is more pronounced in D0_RNA_ populations, suggesting the presence of subpopulations at the pluripotent state, consistent with previous reports^48,49^. The observed disparities could be attributed to the difference in stabilities and half-lives of RNA and protein. By comparison, RNA has a relatively shorter half-life and is more susceptible to degradation than proteins^50^. In turn, it directly impacts the RNA abundance and leads to temporal variations during sampling. In contrast, long protein half-lives and greater stability have led to more consistent expression levels, which can accurately reflect the cellular states and functions.

Overall, these findings demonstrate the ability of single-cell PAGE-PISA to provide a direct and robust depiction of developmental states during dynamic processes, even with minimal sample sizes. Its ability to resolve developmental trajectories from as few as 49 cells not only is particularly promising for identifying subpopulations in samples with limited availability, but also opens up new avenues in various research fields, including developmental biology, regenerative medicine, and disease modeling. Together, single-cell PAGE-PISA serves as a powerful complementary approach to scRNA-seq by bridging the gaps between transcriptome and proteome and providing a more comprehensive understanding of cellular dynamics at the single-cell level.

## Discussion

In this present work, we introduced a single-cell PAGE-PISA capable of highly sensitive and quantitative profiling of a wide range of proteins in individual cells. This system was realized through the integration of PAGE for protein separation based on molecular sizes with 3D single-molecule imaging using PISA for protein quantification. The workflow is streamlined and benchtop-compatible, using microliter-scale sample volumes in standard PCR tubes that can be easily handled with pipettes, eliminating the need for specialized robotic systems.

With single-cell PAGE-PISA, we detected 21 protein bands per single cell and quantified 10^7^ molecules covering approximately 1% of the total proteome per cell. This proportion can be further improved to over 10% by using brighter dyes such as SeTau-647^51^ or advanced spot-recognition algorithms, including deep learning^52^ (Supplementary Fig. 11). With further improvements in separation resolution and labeling efficiency, we anticipate achieving detection sensitivity down to 10^2^–10^3^ molecules per species, enabling the analysis of low-abundance proteins.

A key advantage of single-cell PAGE-PISA is its ability to resolve biologically meaningful subpopulations that are not easily distinguishable using conventional markers. This is particularly important for heterogeneous cell populations such as T cells, which, despite being traditionally viewed as homogeneous, have been shown to comprise distinct subgroups with diverse functional roles^53^. By capturing the full proteomic content of individual cells, such functionally distinct states can be identified. In this study, although a current throughput (*n* < 100) precludes adequate sampling of low-frequency subpopulations, single-cell PAGE-PISA nonetheless resolves biologically meaningful subpopulations, as reflected by the clear separation of precursor, early, and late cardiomyocyte clusters. These subpopulations are also observed in the single-cell transcriptome data, providing independent confirmation that the identified clusters reflect genuine cellular states. Another strength is its ability to resolve modification-specific and structural proteoforms that are often inaccessible to MS-based approaches due to sample loss, limited enrichment efficiency, or disruption of protein complexes during sample preparation. This is enabled by integrating Phos-tag PAGE^54^ or native PAGE, which permits direct detection of phosphorylated isoforms and native protein complexes at the single-cell level. This information is critical for understanding cellular decisions governed by signaling assemblies such as TNF receptor complexes^55^. Furthermore, by labeling specific proteins of interest in situ, this platform can be extended to spatially resolved subcellular proteomics, enabling the analysis of organelle-associated proteins while preserving intracellular localization.

Although chip-based electrophoresis, as implemented in single-cell Western blotting^26^, and capillary electrophoresis (CE) can, in principle, be adapted for proteome analysis using PISA, both approaches face significant limitations for single-cell proteome profiling. The format used in single-cell Western offers high-throughput analysis of over 1000 cells but suffers from limited separation resolution due to its short migration distance (~1 mm), resulting in overlapping bands and reduced quantification accuracy. CE systems, such as the PA 800 Plus (Sciex), can provide higher-resolution separation due to longer capillary lengths. However, their workflows are inherently sequential and typically require 1–2 h per cell, which imposes a major constraint on throughput. Taken together, single-cell PAGE-PISA offers a practical and effective balance of resolution, sensitivity, and throughput for comprehensive proteome profiling at the single-cell level.

Although single-cell PAGE-PISA currently requires approximately 7 h to analyze 20 cells simultaneously, scaling up to thousands of cells will likely be necessary to address broader biological questions. We anticipate that such expansion will be feasible through the use of automated single-cell collection (e.g., fluorescence-activated cell sorting or automated live imaging and picking systems such as ALPS^56^), high-throughput robotic reagent handling, and rapid, parallel electrophoresis (e.g., 10-min runs at 400 V using bullet PAGE from Nacalai Tesque). Although imaging currently limits the overall throughput, our preliminary tests demonstrate that scan speeds can be increased up to 400 µm/s without loss of accuracy^29^. The integration of high-speed CMOS cameras with larger fields of view could further accelerate data acquisition. Overall, scaling up to thousands of cells is estimated to require approximately 30 h from cell isolation to PISA imaging. Since single-cell PAGE-PISA uses only commercially available materials, the cost per cell is estimated between $0.60 and 1.00 and can be reduced to ~$0.11 using homemade gels, making it a highly cost-effective alternative to other single-cell proteomics methods.

While single-cell PAGE-PISA does not yet provide direct molecular identification of protein bands, we have demonstrated that integrating single-cell PAGE-PISA data with complementary omics and immunofluorescence measurements enables inference of candidate proteins within specific molecular weight regions. In this study, protein band changes in the 54.4–58.4 kDa range were cross-validated using transcriptomic, proteomic, and immunofluorescence analyses, linking them to mitochondrial activity and endoplasmic reticulum proteostasis during cardiomyocyte differentiation. Although this approach does not provide definitive identification, it allows for biologically meaningful interpretation and identification of key contributors. More broadly, single-cell PAGE-PISA serves as a practical functional screening tool to monitor proteome changes, generate testable hypotheses, and identify molecular weight regions warranting detailed investigation. Further improvements could involve incorporating multiplexed protein identification directly within the gel using antibodies or multicolor tags, similar to In-Gel Western^57^. Additionally, combining single-cell PAGE-PISA with fluorescent antibody-based sorting via FACS and conventional MS can help associate single-cell proteome profiles with specific protein identities. This integrative approach enables molecular annotation of phenotypically meaningful bands and enhances the ability to interpret cell state differences observed in single-cell PAGE-PISA (Supplementary Table 2).

In conclusion, we have established single-cell PAGE-PISA and demonstrated its potential for ultra-sensitive single-cell proteome analysis on diverse cell types, from steady-state to differentiated cells. We believe that our system can help researchers narrow down the number of potential protein species in each band at the single-cell level prior to immunofluorescent imaging or MS. By focusing on a smaller subset of proteins, this approach not only will help to reduce the time and resources required for downstream analysis but also minimize the need for extensive antibody libraries, making target protein identification more cost-effective for future biomarker discovery.

## Method

### Cell culture

HeLa (RCB0007) and PC-3 (RCB2145) cells were obtained from the RIKEN cell bank. U2OS (HTB-96) cells were purchased from ATCC. The U2OS and HeLa cells were maintained in Dulbecco’s modified eagle medium (DMEM) (Thermo Fisher Scientific, 10566-016) supplemented with 10% fetal bovine serum (FBS) (Corning, 35-079-CV). The PC-3 cells were maintained in RPMI 1640 medium (Thermo Fisher Scientific, 11875-093) supplemented with 10% FBS. Human induced pluripotent stem cells (hiPSCs) were purchased from RIKEN BRC (HPS4290:201B7-Ff) and maintained in mTeSR^TM^ Plus medium (STEMCELL Technologies, 100-0276). All cell lines were maintained in a 5% CO_2_ incubator at 37 °C.

### Cardiomyocyte differentiation protocol

Cardiomyocyte-directed differentiation from hiPSCs was performed and modified based on the previous protocol^58^. Four days before cardiomyocyte differentiation, the hiPSCs were dissociated using TrypLE^TM^ Select CTS^TM^ (Gibco, A12859-01) and cultured in a 24-well plate coated with iMatrix-511 (Nippi, Incorporated, 892011) at a density of 5 × 10^4^ cells/well in mTeSR^TM^ Plus. On day 0, the hiPSCs were washed with phosphate-buffered saline (PBS) (Nacalai-tesque, 14249-95) and treated with differentiation medium (RPMI 1640, GlutaMAX™ (Gibco, 61870036) containing 500 µg/mL human serum albumin (HSA) (Wako, 010-27601) and 213 µg/mL L-ascorbic acid 2-phosphate (Nacalai-tesque, 13571-56)) supplemented with 6 μM CHIR99021 (Wako, 252917-06-9). On day 2, the medium was replaced with fresh differentiation medium supplemented with 5 μM IWP-2 (Wako, 686770-61-6). On day 4, the medium was replaced with a fresh differentiation medium without supplemental inhibitors. Medium exchange was performed every two days. Beating was first observed on day 7. hiPSC-derived cardiomyocytes (hiPSC-CMs) were gently dissociated and transferred to fresh iMatrix-coated plates between days 10 and 12 to improve purity in culture.

### Bulk cell lysate sample preparation

Cells were harvested, washed with PBS three times, and counted using an automatic cell counter (TC20, BioRad) to obtain a concentration of 1.5 × 10^3^ cells/µL. Approximately 50 µL of lysis buffer (50 mM borate (Nacalai-tesque), 1% Tween 20 (Sigma-Aldrich, 9005-64-5), 1% sodium dodecyl sulfate (SDS) (Wako, 192-13981), adjusted at pH 8.0) and 5 µL of protease inhibitor (Nacalai-tesque, 25955) were added to 50 µL of cell suspension. After cell lysis, proteins were labeled with Cy5-NHS ester dye (AAT Bioquest, 151) to a final concentration of 100 µM and incubated at 15 °C and 2000 rpm for 45 min. The dye-labeled proteins were washed with PBS three times in a centrifugal filter unit (10 K) (Millipore, UFC5010BK) and centrifuged at 10,000 × *g* and 4 °C for 10 min. Approximately 50 µL of protein sample was collected after the final wash and stored at −20 °C.

### Lysate spike-in experiment

Approximately 15,000 cell lysates were spiked with 75 ng, 7.5 ng, and 0.75 ng of purified transferrin (Fujifilm Wako, 205-18084), lysed in the presence of protease inhibitor, and labeled with Cy5-NHS ester dye to a final concentration of 100 µM. The samples were washed with 20 mM borate buffer three times in a centrifugal filter unit (10 K) and centrifuged at 10,000 × *g* and 4 °C for 10 min. For single-molecule imaging, the lysate spike-in samples were diluted to obtain cell lysates containing 500 fg, 50 fg, and 5 fg of transferrin. Negative control was prepared containing only bulk cell lysates without exogenous protein.

### Single-cell isolation

A droplet of cell suspension was deposited onto a glass slide (Matsunami, S1111) that was placed on the stage of an inverted microscope (CKX41, Olympus) equipped with a TOPick 1-cell handling system, composed of a touch panel, micro liquid pump, and controller (YODAKA Co., Ltd). The single cells were manually isolated using a 30 µm G-tip low-binding coated micro-glass needle (YODAKA Co., Ltd) with a minimum handling volume of 100 pL. The isolated single cells were transferred to the PCR cap containing 1 µL of PBS, spun down, and stored at −80 °C.

### Single-cell sample preparation

Manually isolated single cells were lysed on ice with 0.6 µL of lysis buffer and 0.4 µL of protease inhibitor. Proteins were labeled with 0.4 µL of Cy5-NHS ester dye to a final concentration of 1 µM and incubated at 15 °C and 2000 rpm for 45 min. To quench the reactivity of excess unreacted dye, 0.4 µL of Tide Quencher™ 5WS amine (AAT Bioquest, 2076) was added to the protein sample to a final concentration of 10 µM. The quencher was added not only to stop the reactivity of NHS ester but to effectively quench the fluorescence of unreacted Cy5-NHS ester dye. As such, it omits the need for common dye removal methods such as gel filtration or desalting columns, which often result in significant protein loss. For efficient protein labeling, the molar ratio of dye to quencher solution used in this protocol was 1:10. To avoid protein loss, all reagents were dispensed along the walls of the PCR tubes, spun down, and stored at −80 °C. Negative control was prepared similar to the single-cell sample but containing only PBS buffer, lysis buffer, protease inhibitor, dye, and quencher solution.

### SDS-PAGE

Electrophoresis was performed using two different polyacrylamide gels depending on the experimental purposes. For standard gel imaging (Fujifilm, LAS 4000), the dye-labeled protein samples from 7.5 × 10^3^ cell lysates were mixed with 4× SDS sample buffer (240 mM Tris-HCl, 40% glycerol stock (Nacalai-tesque, 17045-65), 8% SDS (Nacalai-tesque, 31606-75), and β-mercaptoethanol (Nacalai tesque, 21438-82), adjusted at pH 6.8), heated at 95 °C for 2 min, and separated using a 12-well, 5‒20% precast polyacrylamide gel (Bio-craft, #SDG-571). The loading volume was 12 µL. For single-molecule imaging, the dye-labeled protein samples were prepared, heated at 95 °C for 2 min, and separated using a 20-well, 5‒20% precast polyacrylamide gel (Bio-craft, #SDG-576). A total volume of 8 µL was loaded for highly diluted bulk cell lysates equivalent to single-cell level and 5 µL for single-cell lysates, both containing 4× SDS sample buffer. To avoid diffusion of free Cy5-NHS ester dye to the adjacent lanes during protein migration, the samples were applied and separated by two loading wells. All gels were irradiated under UV overnight to remove autofluorescence signals that could contribute to the background noise. Electrophoresis was performed at 250 V and 30 mA for 70 min.

### Single-molecule imaging

After SDS-PAGE, the polyacrylamide gel was cut vertically into a 5 cm length, covering the majority of the protein bands in the middle molecular weight region, and placed on a UV-irradiated fluorinated ethylene propylene (FEP) film (Daikin Chemical, NF-0025). To avoid gel desiccation and reduce the reflection of excitation illumination on the gel surface during observation, two blank polyacrylamide gels were placed on top of the gel with protein bands, followed by a thin layer of transparent film (Supplementary Fig. 1c, d). This configuration allows for a longer PISA observation of up to four hours.

All single-molecule fluorescence imaging in this study was performed by our custom-built light-sheet microscope, namely PISA^29^. The optical configuration of PISA is similar to that of open-top inverted selective plane illumination microscopy^59^, in which both objective lenses are positioned below the coverslip at a tilted angle of 33.8 degrees. PISA was built on a custom microscope body with two orthogonally connected water-immersion objective lenses, fluorescence illumination (Special Optics, 54-10-7, NA = 0.66, 28.6×) and detection (Evident, XLUMPLFLN 20XW, NA = 1.0, 20×). Bessel beam was generated by passing the laser source via an axicon lens (Mie Optics), which was then reflected and scanned along *x*-axis (cf. the perpendicular axis to the electrophoresis migration) by a Galvano mirror (Cambridge Technology, 6215HB), creating a light sheet with a width of 400 µm and typical thickness of 4 µm. The microscopic stage is mounted on a motorized stage (Prior Scientific, H117) for the *xy*-axis translation, which was controlled by a homemade program written in LabVIEW (National Instruments, 2021). The detection port was connected to an EM-CCD camera (Andor, iXon Ultra 897) via an imaging lens and used to image fluorescence single molecules.

Imaging was conducted using a 647 nm fiber laser (MPB Communications, 2RU-VFL-P-2000-647) at 1000 mW and detected through a near-infrared band-pass filter (Semrock, FF01-708/75-25) controlled by a motorized filter wheel (FW102C, Thorlabs, USA). The protein bands embedded in polyacrylamide gel were imaged by scanning the gel along the axis of a migration direction with a 4 μm step size at 50 ms exposure per frame. Images were acquired using a commercial software (Molecular Devices, MetaMorph, version 7.10.3.279) and saved in TIFF format for further image analysis. Typically, scanning of one lane took approximately 10 min.

### Immunofluorescence imaging

iPSCs (D0) and iPSC-CMs (D30) were detached using TrypLE™ Select CTS™ for 10–15 min at 37 °C and replated onto iMatrix-coated µ-slide 8 well chambers (Ibidi, 80826). Cells were chemically fixed with 4% paraformaldehyde for 25 min and permeabilized with 0.1–0.5% Triton X-100 for 5 min. Cells were then blocked with 1% BSA and 0.2% Triton X-100 in PBS and incubated with primary antibodies for one hour at room temperature, followed by incubation with secondary antibody for an additional one hour at room temperature. cTnT and SOX2 were used as positive controls for cardiac and pluripotency markers, respectively. A negative control experiment was performed to verify the absence of non-specific secondary antibody binding in fixed samples. Nuclei were counterstained with DAPI (Ibidi, Cat. No. 50011). *z*-stacks (1 µm intervals) images were acquired using a Zeiss confocal microscope (LSM880 with Airyscan) with a 63× objective. Imaging parameters including laser power and detector gain were kept constant across all samples and conditions to allow direct comparison between D0 and D30. Antibodies are listed in Supplementary Table 3.

### Image analysis

All raw images were opened in ImageJ software (version 1.51n)^60^, pre-processed using the rolling ball algorithm for background subtraction, and imported into Arivis Vision 4D (Zeiss, version 4.0.0) (Supplementary Fig. 5a, b). Denoising filter was applied to the background-subtracted images to improve the signal-to-noise ratio and a blob finder tool was used to detect spots in the 3D image matrix (Supplementary Fig. 5c). Segment filter was optionally applied to remove single molecules with low-intensity signals. The images were saved and the acquired dataset containing the information of each spot, corresponding to single dye-labeled proteins, was exported in CSV format for data analysis.

For immunofluorescence analysis, confocal *z*-stack images were projected into a single plane using sum-intensity projection. Background in the green channel was removed using rolling ball subtraction. Cell segmentation was performed with Cellpose^61^ and the resulting regions of interest (ROIs) were exported for downstream analysis. Partial and multilayered cells were excluded to avoid underestimation of cellular fluorescence. Integrated fluorescence intensity per cell was quantified from the green channel.

## Data analysis

The exported dataset was analyzed and visualized using OriginPro (OriginLab, 2022) and Python (version 3.10.11). The frequency count function was used to generate a binned dataset and provide the count of dye-labeled proteins in each bin. The data was organized into a matrix of protein count × cell ID (rows × columns). To compensate for gel-to-gel variability, the proteome profile of each cell lysate was manually aligned and scaled to standardize the peak (protein bands) positions across multiple technical or biological replicates. Molecular weights of unknown proteins were estimated by fitting a standard curve relating the known molecular weights of protein standards to their migration distance.

To correct for slope differences between proteome profiles, the ratio of protein counts between a reference (either bulk cell lysates or the average of all single-cell lysates) and individual cell lysates was calculated across molecular weights. A linear fit was applied to this ratio to estimate the overall slope, which was then used to correct the protein counts of individual cell lysates. The resulting slope-corrected values are referred to as normalized protein counts and are used for comparisons of protein band expressions. To restore protein abundance information after slope correction, the ratio between the original and normalized protein counts was calculated across molecular weights and the mean ratio was applied globally to the normalized protein counts. The resulting values, which reflect actual protein abundance with slope bias removed, are referred to as adjusted protein counts and are used for comparisons of total protein abundance between cells.

UMAP was used to visualize single cells in two (or three) dimensional maps and the clusters were identified using *k*-means. Diffusion map was used to visualize the cell trajectory during cardiomyocyte differentiation by employing a random-walk algorithm to estimate the cell transition probabilities based on a weighted nearest neighbors graph^40^. Trajectory was inferred by computing diffusion pseudotime (DPT) for each cell relative to the root cell, and the pseudotemporal values were assigned to the cells along the inferred trajectory. Differential protein bands (visualized in volcano plot) were determined by the Welch’s two-sided, two-sample *t*-test (*p* < 0.05, fold change >1.2). For pairwise comparisons between three or more groups, significant differences were determined by the one-way Welch’s ANOVA with Games-Howell post-hoc test (*p* < 0.05). The scRNA-seq datasets for D0, D15, and D30 were obtained from the ArrayExpress database at EMBL-EBI under the accession number E-MTAB-6268^41^. Three random samples (RS) were generated from the dataset, each containing 49 randomly selected single cells from D0, D15, and D30 (*n* = 12, 14, and 23, respectively). For a larger sample size, 3000 single cells were randomly selected from D0, D15, and D30 (*n* = 1000 for each sampling day). Differential genes were determined by the Welch’s two-sided, two-sample *t*-test (*p* < 0.05, fold change >1.4). The analysis included a total of 10,484 genes, which represented all genes corresponding to proteins between 37 and 126 kDa. The protein molecular weights were obtained from UniProt.

### Reporting summary

Further information on research design is available in the Nature Portfolio Reporting Summary linked to this article.

## Supplementary information

Supplementary Information

Description of Additional Supplementary Files

Supplementary Movie 1

Supplementary Movie 2

Reporting Summary

Transparent Peer Review file

## Source data

Source Data

## Data Availability

Data generated in this study are provided in the Source Data file. Source data are provided with this paper.

## Code availability

All computational analyses in this study were performed using standard functions from publicly available Python packages, including scanpy (for UMAP, diffusion map, and diffusion pseudotime analyses), scikit-learn (for *k*-means clustering and PCA), and SciPy (for statistical tests of Spearman correlation). All parameters and procedures used in the analyses, including the slope-correction normalization, are fully described in the Methods section, enabling full reproduction of the results without additional code. No algorithms or substantial custom implementations were developed in this study.
