# Supplementary material for: Molecular profiling of the single-cell proteome via gel electrophoresis and 3D single-molecule imaging
Source: Nat Commun. 2026 Jul 8;17:5727. doi: 10.1038/s41467-026-74840-0 (PMC13346896; doi:10.1038/s41467-026-74840-0)
Supplement: Supplementary file 2 — Description of Additional Supplementary Files [file 41467_2026_74840_MOESM2_ESM.pdf]

Description of Additional Supplementary Files

File Name: Supplementary Movie 1

Description: Single-molecule fluorescence imaging of dye-labelled proteins originating from individual cell lysates separated by polyacrylamide gel electrophoresis. The proteins are then visualized using PISA with 647 nm excitation, where each detected fluorescence spot corresponds to a single dye-labelled protein molecule. Fluctuations in the number of detected molecules along the migration path reflect protein bands with different protein abundances.

File Name: Supplementary Movie 2

Description: Single-molecule fluorescence imaging of the negative control was performed under identical PISA conditions (647 nm excitation) in the absence of single-cell lysate, but in the presence of buffer components including PBS, lysis buffer, Cy5 dye, and quencher. No electrophoretic protein bands are observed and only minimal background signals are detected, confirming that the fluctuations in the molecular number seen in Supplementary Movie 1 are proteins originating from single-cell lysates.
