# Supplementary material for: Molecular profiling of the single-cell proteome via gel electrophoresis and 3D single-molecule imaging
Source: Nat Commun. 2026 Jul 8;17:5727. doi: 10.1038/s41467-026-74840-0 (PMC13346896; doi:10.1038/s41467-026-74840-0)
Supplement: Supplementary file 5 — Reporting Summary [file 41467_2026_74840_MOESM5_ESM.pdf]

nature portfolio

Corresponding author(s): Yuichi Taniguchi

Last updated by author(s): May 21, 2026

Reporting Summary

Nature Portfolio wishes to improve the reproducibility of the work that we publish. This form provides structure for consistency and transparency in reporting. For further information on Nature Portfolio policies, see our Editorial Policies and the Editorial Policy Checklist.

Statistics

For all statistical analyses, confirm that the following items are present in the figure legend, table legend, main text, or Methods section.

n/a	Confirmed	
☐	☒	The exact sample size (*n*) for each experimental group/condition, given as a discrete number and unit of measurement
☐	☒	A statement on whether measurements were taken from distinct samples or whether the same sample was measured repeatedly
☐	☒	The statistical test(s) used AND whether they are one- or two-sided<br>*Only common tests should be described solely by name; describe more complex techniques in the Methods section.*
☒	☐	A description of all covariates tested
☒	☐	A description of any assumptions or corrections, such as tests of normality and adjustment for multiple comparisons
☐	☒	A full description of the statistical parameters including central tendency (e.g. means) or other basic estimates (e.g. regression coefficient) AND variation (e.g. standard deviation) or associated estimates of uncertainty (e.g. confidence intervals)
☐	☒	For null hypothesis testing, the test statistic (e.g. *F*, *t*, *r*) with confidence intervals, effect sizes, degrees of freedom and *P* value noted<br>*Give P values as exact values whenever suitable.*
☒	☐	For Bayesian analysis, information on the choice of priors and Markov chain Monte Carlo settings
☒	☐	For hierarchical and complex designs, identification of the appropriate level for tests and full reporting of outcomes
☒	☐	Estimates of effect sizes (e.g. Cohen's *d*, Pearson's *r*), indicating how they were calculated

Our web collection on statistics for biologists contains articles on many of the points above.

Software and code

Policy information about availability of computer code

Data collection	3D single-molecule imaging data was performed using our recently developed PISA microscope (Taniguchi et al., 2024). Images were acquired using a commercial software (Molecular Devices, MetaMorph, version 7.10.3.279) and a motorized stage (Prior Scientific, H117), which were controlled by a homemade program written in LabVIEW (National Instruments, 2021) and saved in TIFF format for image analysis. Images were preprocessed using ImageJ software (version 1.51n) and imported into Arivis Vision 4D (Zeiss, version 4.0.0) for spot detection. The images were saved and the acquired dataset containing the information of each spot, corresponding to single dye-labelled proteins, was exported in CSV format for data analysis.
Data analysis	Data analysis was performed using OriginPro (Originlab, 2022) and Python (version 3.10.11). All computational analyses in this study were performed using standard functions from publicly available Python packages, including scanpy (for UMAP, diffusion map, and diffusion pseudotime analyses), scikit-learn (for k-means clustering and PCA), and SciPy (for statistical tests of Spearman correlation). All parameters and procedures used in the analyses, including the slope-correction normalization, are fully described in the Methods section, enabling full reproduction of the results without additional code. No algorithms or substantial custom implementations were developed in this study.

For manuscripts utilizing custom algorithms or software that are central to the research but not yet described in published literature, software must be made available to editors and reviewers. We strongly encourage code deposition in a community repository (e.g. GitHub). See the Nature Portfolio guidelines for submitting code & software for further information.

Data

Policy information about availability of data

All manuscripts must include a data availability statement. This statement should provide the following information, where applicable:

- Accession codes, unique identifiers, or web links for publicly available datasets
- A description of any restrictions on data availability
- For clinical datasets or third party data, please ensure that the statement adheres to our policy

Source Data are provided with this paper

Research involving human participants, their data, or biological material

Policy information about studies with human participants or human data. See also policy information about sex, gender (identity/presentation), and sexual orientation and race, ethnicity and racism.

Reporting on sex and gender	This study did not involve human participants. Sex/gender of the donor for the established hiPSC line (HPS4290:201B7-Ff) is not relevant to the methodological scope of this study.
Reporting on race, ethnicity, or other socially relevant groupings	This study did not involve human participants. Donor race/ethnicity for the established hiPSC line is not relevant to the methodological scope of this study.
Population characteristics	This study did not involve human participants. The hiPSC line used (HPS4290:201B7-Ff) was obtained from RIKEN BRC.
Recruitment	No human participants were recruited in this study. The hiPSC line was obtained from RIKEN BRC under their established framework.
Ethics oversight	No new human participants were recruited and no primary human tissue was obtained for this study. The hiPSC line HPS4290:201B7-Ff was obtained from RIKEN BRC, which manages donor consent and ethical oversight for distribution of the line. No additional ethics board approval was required at our institution for the use of this established commercially available cell line.

Note that full information on the approval of the study protocol must also be provided in the manuscript.

Field-specific reporting

Please select the one below that is the best fit for your research. If you are not sure, read the appropriate sections before making your selection.

☒ Life sciences ☐ Behavioural & social sciences ☐ Ecological, evolutionary & environmental sciences

For a reference copy of the document with all sections, see nature.com/documents/nr-reporting-summary-flat.pdf

Life sciences study design

All studies must disclose on these points even when the disclosure is negative.

Sample size	No statistical methods were used to predetermine sample size for the experiments in this study. Sample sizes were determined based on the number of successfully collected and analyzable single cells obtained after manual single-cell isolation, image acquisition, and quality-control filtering to exclude low-quality images or failed measurements. For method development and demonstration, we analyzed 10 single cells each from PC-3 and U2OS cell lines. To showcase the applicability and versatility of our method, we analyzed 12, 14, and 23 single cells from hiPSCs, hiPSC-CMs (D16), and hiPSC-CMs (D30), respectively. These sample sizes were considered sufficient, as protein band detection was reproducible and clear differences between cell types and differentiation stages were observed across the analyzed cells.
Data exclusions	Images that were of low quality due to high background signals or artifacts were excluded from the dataset.
Replication	Experiments were performed independently and repeatedly over a period of approximately 6 months, with imaging typically conducted 1–2 times per week. Each experiment involved independent manual single-cell isolation, sample preparation, imaging, and downstream analysis. Experimental sessions included either measurements of a single cell type or differentiation stage, or parallel measurements of multiple cell types or stages for comparison.
Randomization	Not relevant for the single-cell proteome studies as only cultured cells were used for method demonstration.<br>We obtained scRNA-seq datasets for D0, D15, and D30 from the ArrayExpress database (EMBL-EBI, accession number E-MTAB-626839). To ensure unbiased sampling, we generated three random samples, each composed of 49 randomly selected single cells across the three time points (D0: n=12, D15: n=14, D30: n=23) from the entire dataset. For a more comprehensive analysis, we also randomly selected a larger sample of 3,000 single cells, with 1,000 cells from each time point (D0, D15, D30).
Blinding	Blinding is not relevant for this study as it is a method of development and demonstration.

Reporting for specific materials, systems and methods

We require information from authors about some types of materials, experimental systems and methods used in many studies. Here, indicate whether each material, system or method listed is relevant to your study. If you are not sure if a list item applies to your research, read the appropriate section before selecting a response.

Materials & experimental systems

n/a	Involved in the study
☐	☒ Antibodies
☐	☒ Eukaryotic cell lines
☒	☐ Palaeontology and archaeology
☒	☐ Animals and other organisms
☒	☐ Clinical data
☒	☐ Dual use research of concern
☒	☐ Plants

Methods

n/a	Involved in the study
☒	☐ ChIP-seq
☒	☐ Flow cytometry
☒	☐ MRI-based neuroimaging

Antibodies

Antibodies used	Anti-ATP5B antibody (mouse IgG), supplied by Abcam (catalog no. ab14730); lot no. 2101066744; clone: 3D5, dilution factor 1:1000.<br>Anti-P4HB antibody (mouse IgG), supplied by Abcam (catalog no. ab2792); lot no. 1103732-5; clone: RL90, dilution factor 1:1000.<br>Anti-MT-CO1 antibody (mouse IgG), supplied by Abcam (catalog no. ab14705); lot no. 1084743-43; clone: 1D6E1A8, dilution factor 1:100.<br>Anti-cTnT antibody (mouse IgG), supplied by Abcam (catalog no. ab8295); lot no. 1029723-3; clone: 1C113, dilution factor 1:1000.<br>Anti-SOX2 antibody (mouse IgG), supplied by Abcam (catalog no. ab79351); lot no. 1107587-4; clone: 9-9-3, dilution factor 1:200.<br>Anti-PHGDH antibody (mouse IgG), supplied by Sigma-Aldrich (catalog no. WH0026227M1); lot no. LB081-S2; clone: 4A3-1D6, dilution factor 1:333.<br>Anti-PDIA3 antibody (mouse IgG), supplied by Abcam (catalog no. ab13506); lot no. 1022455-12; clone: Map.ERP57, dilution factor 1:100.<br>Anti-mouse IgG secondary antibody (Alexa Fluor 488) (goat IgG), supplied by Thermo Fisher Scientific (catalog no. A11001); lot no. 2833436; clone: not applicable (polyclonal), dilution factor 1:2000.
Validation	All primary antibodies used in this study were commercially sourced and validated by the manufacturers for applications including immunofluorescence, as indicated on the respective product datasheets. Antibody specificity was further supported by detection of signals at expected molecular weights and consistent expression patterns across experiments. Positive controls were performed using cTnT as a cardiac marker and SOX2 as a pluripotency marker. Negative controls were performed using secondary antibodies only (without primary antibodies) to assess the non-specific binding.

Eukaryotic cell lines

Policy information about cell lines and Sex and Gender in Research

Cell line source(s)	HeLa (RCB0007), PC-3 (RCB2145), and iPSCs (HPS4290:201B7-Ff) were purchased from RIKEN BRC; U2OS (HTB-96) was purchased from ATCC.
Authentication	No additional authentication was performed on these cell lines beyond the authentication conducted by the supplying cell banks (RIKEN BRC and ATCC) prior to distribution.
Mycoplasma contamination	All cell lines were tested negative for mycoplasma contamination.
Commonly misidentified lines (See ICLAC register)	No misidentified cell lines were used.

Plants

Seed stocks	N/A
Novel plant genotypes	N/A
Authentication	N/A
