# Supplementary material for: Molecular profiling of the single-cell proteome via gel electrophoresis and 3D single-molecule imaging
Source: Nat Commun. 2026 Jul 8;17:5727. doi: 10.1038/s41467-026-74840-0 (PMC13346896; doi:10.1038/s41467-026-74840-0)
Supplement: Supplementary file 6 — Transparent Peer Review file [file 41467_2026_74840_MOESM6_ESM.pdf]

nature portfolio

Peer Review File

Molecular profiling of the single-cell proteome via gel electrophoresis and 3D single-molecule imaging

Corresponding Author: Professor Yuichi Taniguchi

This file contains all reviewer reports in order by version, followed by all author rebuttals in order by version.

Version 0:

Reviewer comments:

Reviewer #1

(Remarks to the Author)
I liked this paper and think that it’s worth publishing with minor modifications, even though the technology described is likely a dead end.

It’s a dead end for the following reasons:

1. What PAGE-PISA provides is a low-resolution trace (e.g., Figure 2e) that, by my estimate, has a mass resolving power of 2-4 kDa (FWHM) for 3 or 4 more or less resolved peaks. The rest of the trace is a fused envelope. The total number of “features” that are statistically significant on a given trace is probably 10 or so.

2. Even the resolved peaks are probably multiplets. Thus the difference between two proteomes cannot be attributed to a difference in abundance of one or even several proteins; massive shifts in protein abundance result in a small variation of the overall envelope. Even when the authors can see a shift at a certain MW, they are uncertain what protein this can be attributed to. An example they provide on p. 10 where they wildly speculate, but unable to tell for sure, the protein identity, is a case in point.

3. In contrast, mass spectrometry based single cell proteomics (MS-SCP) can identify and quantify robustly 2000 proteins and with less certainty – up to 5,000 proteins per cell. There is no way that PAGE-PISA can approach these numbers even theoretically.

Still, as said, I liked this paper, because:

1. It’s a daring enterprise where, using commercial parts (e.g., 8 cm PAGE) clearly not designed to handle such small sample volumes, one achieves spectacular results and proves beyond reasonable doubts that these results are real.

2. The authors provide a spectacular range of ideas how to improve their technology. Even though I don’t think any of this will help PAGE-PISA to become competitive with MS-SCP, it demonstrates their ingenuity and creativity.

What I didn’t like, however, is the overselling PAGE-PISA. The authors should be more realistic and provide data on the peak capacity of their “spectra” as well as the mass resolution. They should also acknowledge that PAGE-PISA is no match to MS-SCP and unlikely to be ever.

I was also put down by the fact that on Figure 2f the UMAP analysis separated two cell lines only in the 3d, least significant, dimension. Even though am not surprised, for the authors it should have been a wake-up call demonstrating that their technique produces barely useable data.

But as said, if the authors provide a more sober account of their technique, they will deliver an interesting read and an inspiration for great many scientists.

Reviewer #2

(Remarks to the Author)
Comments for authors

Kamarulzaman and coworkers here apply a method they described in a preprint that allows bands to be measured after polyacrylamide gel electrophoresis (PAGE) of single cells. The authors use an N-hydroxysuccinimide (NHS) ester dye to label all proteins and a planar illumination microscope for single molecule imaging for all purposes (PISA, described at https://doi.org/10.1101/2024.12.05.625331) for detection. They applied their method to U2OS and PC-3 cells and compared their single-cell proteome with bulk data, demonstrating to some extent (although not thoroughly) the possibility of performing single-cell measurements. The authors also applied their method to cardiomyocyte differentiation using human induced pluripotent stem cells (hiPSCs).

While the ability to observe bands for individual cells has some appeal, and we truly believe that the technology could lead to some interesting applications in the future, the manuscript suffers from conceptual and methodological flaws that in our opinion make it not suited for Nature Communications.
The use of the term "single-cell proteomics" is misleading because the methodology described does not identify proteins or quantify them with the depth and accuracy of MS-based single-cell proteomics, which is the established gold standard in the field. Instead, it relies on PAGE profiling to provide proteome "fingerprints" that lack molecular identity information and provide no compelling evidence of how this can provide useful new information beyond existing fluorescence imaging or FACS methods.
It is poorly clarified how useful the method will be and for which applications it will offer advantages over existing technologies, particularly MS-based single-cell proteomics. Claims of sensitivity to low-abundance proteins are not supported by robust experimental evidence, and critical controls such as spiked-in proteins or dilution series to validate reproducibility and sensitivity are lacking. Furthermore, the throughput of the method appears to be low, and the possibility of scaling up to thousands of cells or identifying rare subpopulations is not addressed.
The manuscript also relies heavily on an unpublished previous work, the PISA method, where essential details regarding sensitivity and validation have not been evaluated. This study lacks a basic background that would allow a strong independent application of the methodology.
The experimental design and data presentation do not allow confidence in the reliability or significance of the results. Basic control experiments, such as comparing single cell and bulk lysates or loading multiple single cells to establish linearity, were not performed, further weakening the claims made in this study. In our opinion this work needs to be extensively revised to be of interest for this journal, and more data presented to prove its value for single cell analysis.

Major points:

1. Throughout the paper, the term "single-cell proteomics" is used in a misleading way. What the authors here call "single cell proteomics" is rather the profiling of the major bands after PAGE for what appear to be single cells. The authors should find a more appropriate and less misleading term since there are MS-based single-cell proteomics technologies that provide protein identities for several hundred species and relative levels for several of them in single isolated cells.

2. The authors acknowledge that MS-based single-cell proteomics technologies exist and that they are capable of detecting 10^4 copies per cell. One aspect that they misleadingly present is that these technologies also provide protein identity information, which is crucial for comparing the two methods. This calls into question the usefulness of their method, which provides a "fingerprint" for each cell, but not protein identity information.

3. Related to the previous point, although the method could be used to distinguish different cells in a population, the approach seems extremely laborious for this purpose. In other words, simple staining for a few cell markers would provide this type of information (either by microscopy or FACS). The authors could claim that "fingerprinting" the proteome would provide a more reliable way to cluster different cell types, but no application is actually provided, making the utility of this method rather difficult to understand. Can the authors speculate on the practical usefulness of their method?

4. At the current stage we obviously see the importance of the technological development of the PISA method (https://doi.org/10.1101/2024.12.05.625331) as an interesting technical application, but the current paper should stand alone. Although it has a lot of potential and we think a little more work could make it very interesting, at this stage we are struggling to understand its power in the context of cell phenotyping. One way to demonstrate the use of the method is that in direct comparison with MS-based single cell proteomics, it has some advantages (throughput? Real sensitivity in both identification and measurement of low abundance proteins? etc.). This is not the case in its current form. For example, there is no indication of protein identities and no indication that the method can actually identify (with their molecular identity) low abundance proteins as suggested. Can the authors measure the identity of different bands from the bulk and use this as a possible way to interpret at least some phenotypic changes in the single cell environment? We would find a similar application potentially interesting and at least could be more directly compared and benchmarked with MS-based single cell proteomics technologies, possibly providing a reason why this method would be preferable.

5. Phenotypically, the differences during cardiomyocyte differentiation are very large, and fluorescence imaging or simply staining for a few proteins such as MyoD and myosins will provide very similar information for thousands of cells, providing equally reliable staging and grouping of cells. We think the most interesting part of the work would be to identify a subpopulation of cells that are not easily distinguishable, or alternatively, to provide real molecular information about the bands that are identified (which is missing in this work). This is now possible with MS-based single cell proteomics. Can the authors provide an application where this method would outperform MS-based single cell proteomics? We think this would

be possible, for example, if it also quantifies aspects that are more difficult to measure with MS-based single-cell proteomics technologies, such as specific protein modifications or protein complexes (in non-denaturing gels) that are not easily seen with these technologies. Overall, we think that the authors should find an obvious application that makes this method useful and allows to measure or identify something that could not be seen or measured at the single cell level before.

6. Figure 4 from the PISA method and the description of the method itself (https://doi.org/10.1101/2024.12.05.625331), which are not yet published, are important to provide information about the sensitivity of the method. Similarly, the description of the details of the imaging method is not peer-reviewed per se. The authors may be in the process of publishing the original paper, but if this is not the case, the current paper would be missing an important set of information and validations, so we feel somewhat confused as we see this paper as a continuation of a previous work that may have clear limitations in its unpublished form and may never be published.

7. One question is about a central point that is also importantly raised by the authors and that remains. Rare subpopulations of cells with distinct properties are often masked at the bulk level, but the throughput proposed by the authors is relatively low, and the authors do not use their method to identify rare populations. What is the current throughput of this method? Would it be possible to scale this up to thousands of cells? If so, at what cost per cell and how long would it take to do so? The authors should discuss at least some of these aspects more openly and possibly refocus the introduction (see minor points).

8. The reproducibility of running small amounts for bulk lysates that "simulate" single cell concentration is largely biased by the more intense bands (more abundant proteins), but one of the larger claims of the authors is the ability to see and detect very low abundance proteins. The authors should design a "lysate spike-in experiment" to show that spiking even small amounts of an exogenous protein (GFP?) into the complex cellular mixture can indeed provide reliable detection of low abundance proteins as also defined by the authors (10^2-10^3). In doing so, the authors should adjust the total fluorescence so that the band of the exogenous protein is likely to be smaller than the ~100 more abundant proteins, all of which are likely to be more abundant than 10^4. Considering the blurred signal in Fig. 2b, we don't think a protein in this range can be visualized at all, as it would probably be difficult to see it in a normal SDS-PAGE where the signal comes from thousands of cells (similar to Supp. Fig. 2a).

9. We are confused by Supp. Fig. 3: are diluted bulk also run in parallel with single cells? If not, this should be added to show that the dilution is indeed reliable (experiments in Supp. Fig. 2 are reliable). Similarly, to show the true ability of the method to linearly discriminate the intensity of single cells, an experiment with 1, 2, 4 and 8 cells should be performed.

Minor points:

1. The title is misleading. As single-cell proteomics is becoming a reality, such a paper should have a title that more accurately reflects the actual content and clearly states that the main topic is the development of a single-cell PAGE approach for the analysis of protein bands after N-hydroxysuccinimide-ester dye staining.

2. The introduction and especially the first paragraph lack focus and should better reflect the actual strength of the work, which will probably require a more careful refocusing based on the actual applications (see main points).

3. The authors correctly point out that several other groups have developed single-cell Western blots. Likewise, capillary electrophoresis can be used for similar approaches. Can the authors reliably compare their method with modifications of these methods tailored to see the whole proteome (at similar NHS conjugations)? In other words, here the method seems particularly interesting for sensitivity, but "whole proteome visualization" using N-hydroxysuccinimide-ester dye (or other protocols) is not innovative, and several other solutions provide this type of signal. Can the authors provide some sort of comparison with one of these other single cell systems, adapted for the purpose of imaging all proteins, to benchmark their sensitivity? We think this would be helpful to show the superior sensitivity of their method compared to other methods.

4. Which statistical test was used in Figure 2d, 3d, and 3i? The authors should mention this in the figure legend.

Reviewer #3

(Remarks to the Author)
I co-reviewed this manuscript with one of the reviewers who provided the listed reports. This is part of the Nature Communications initiative to facilitate training in peer review and to provide appropriate recognition for Early Career Researchers who co-review manuscripts.

Version 1:

Reviewer comments:

Reviewer #1

(Remarks to the Author)
Am satisfied with the response by the authors and wish the manuscript good luck

Reviewer #2

(Remarks to the Author)
We thank the authors for their diligent and thoughtful revisions. The technical quality of what is performed is clearly visible, and the method is described with the necessary detail.

Nevertheless, in our opinion, several important issues remain unanswered.

The most critical issue is related to the inconsistency between the theoretical potential of the method and its demonstrated utility. In the absence of molecular identification of the fingerprinted proteins, in our opinion the data remain difficult to interpret in biological terms.

The claims of sensitivity (particularly for low-abundance proteins) are not well supported, as clear in the spike-in experiment in Supplementary Figure 3, where linearity is lost and the middle lane shows no visible signal. In addition, claims of detection of rare populations or minor phenotypes remain to be confirmed without benchmarking and comparison against other established single-cell approaches, including imaging ones.

That being said, we do recognize that the research is well done technically and that its novelty and interest for the community are editorial judgments. We leave that to the discretion of the editor in this case and agree with their final decision.

We want to thank the authors for their efforts and hope these comments are useful as they continue to refine and develop their method. We look forward to a future improvement to highlight the strength of this approach for aspects that cannot be solved with other methods.

Reviewer #3

(Remarks to the Author)
I co-reviewed this manuscript with one of the reviewers who provided the listed reports. This is part of the Nature Communications initiative to facilitate training in peer review and to provide appropriate recognition for Early Career Researchers who co-review manuscripts.

Version 2:

Reviewer comments:

Reviewer #2

(Remarks to the Author)
The authors have made a substantial effort to address the reviewer's comments, and the revised manuscript reflects a clear improvement in both technical rigor and clarity. The additional experiments, particularly the immunofluorescence validation and the revised spike-in analysis, strengthen the methodological framework and provide greater confidence in the robustness of the approach.

The authors now offer a more transparent and realistic assessment of the method's capabilities. The integration of single-cell PAGE-PISA with complementary datasets (single-cell transcriptomics, proteomics, and imaging) represents a thoughtful strategy to enhance biological interpretability in the absence of direct protein identification. While this remains an indirect approach, it is applied carefully and yields biologically coherent insights, particularly in the context of cardiomyocyte differentiation.

The revised sensitivity analysis and normalization procedure are technically sound. Although the detection limits and throughput do not yet match those of cutting-edge single-cell proteomics platforms, the method is now presented in a more balanced and credible way. Similarly, the authors appropriately moderate their claims regarding rare population detection and provide orthogonal validation supporting the observed cellular heterogeneity.

Overall, the work is technically solid.

Reviewer #3

(Remarks to the Author)
I co-reviewed this manuscript with one of the reviewers who provided the listed reports. This is part of the Nature Communications initiative to facilitate training in peer review and to provide appropriate recognition for Early Career Researchers who co-review manuscripts.

Open Access This Peer Review File is licensed under a Creative Commons Attribution 4.0 International License, which permits use, sharing, adaptation, distribution and reproduction in any medium or format, as long as you give appropriate credit to the original author(s) and the source, provide a link to the Creative Commons license, and indicate if changes were made.
In cases where reviewers are anonymous, credit should be given to 'Anonymous Referee' and the source.
The images or other third party material in this Peer Review File are included in the article's Creative Commons license, unless indicated otherwise in a credit line to the material. If material is not included in the article's Creative Commons license and your intended use is not permitted by statutory regulation or exceeds the permitted use, you will need to obtain permission directly from the copyright holder.
To view a copy of this license, visit https://creativecommons.org/licenses/by/4.0/

Response to the reviewers' comments:

<u>Our response to Reviewer #1:</u>

We sincerely appreciate Reviewer #1 for the insightful comments that have helped improve the quality of our paper. As outlined in the following responses, we have carefully considered all comments in our revision. The reviewer's remarks are presented in ***bold italics***, followed by our responses. Modifications in the revised manuscript are highlighted in red font.

I liked this paper and think that it's worth publishing with minor modifications, even though the technology described is likely a dead end.

It's a dead end for the following reasons:

1. What PAGE-PISA provides is a low-resolution trace (e.g., Figure 2e) that, by my estimate, has a mass resolving power of 2-4 kDa (FWHM) for 3 or 4 more or less resolved peaks. The rest of the trace is a fused envelope. The total number of "features" that are statistically significant on a given trace is probably 10 or so.

We thank the reviewer for the comment. To address this concern, we conducted a detailed peak fitting analysis of a representative single-cell electropherogram to characterize the mass resolution and feature capacity of single-cell PAGE-PISA. We identified 21 distinct Gaussian peaks between 35 and 128 kDa in a representative single-cell proteome profile (Figure 1a). For proteins in the 35–60 kDa range (peaks #1–8), the average mass resolution was 1.34 kDa (FWHM). For proteins in the 60–128 kDa range (peaks #9–21), the resolution declined to an average of 2.69 kDa, likely due to reduced gel migration efficiency for large proteins (Figure 1b). We have added these data in Supplementary Fig. 6a and 6b and discussed the results in lines 205–210, page 8.

a

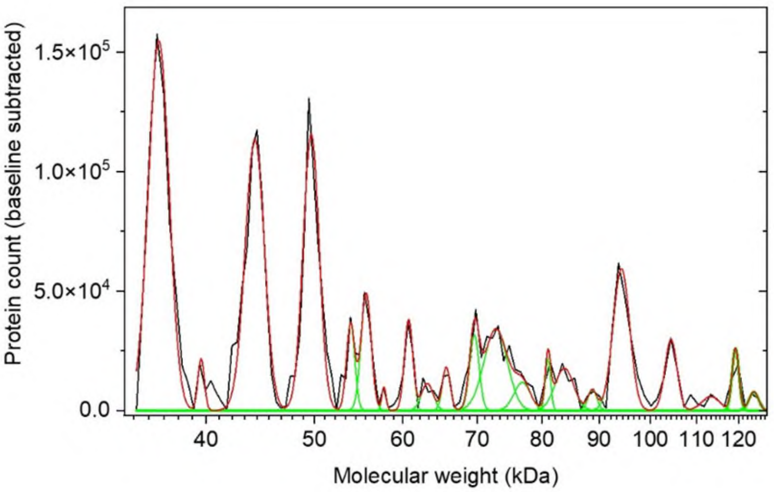

b

Peak Index	Peak Type	Area Intg	FWHM	Max Height	Molecular weight	Area IntgP
1	Gaussian	294758.8118	1.82357	154559.35947	36.27853	16.3423
2	Gaussian	14172.53824	0.61584	21619.43885	39.59333	0.78577
3	Gaussian	248586.55248	2.05845	113450.16798	44.22884	13.78237
4	Gaussian	235827.457	1.91745	115541.69403	49.68373	13.07497
5	Gaussian	41984.88533	1.08574	36327.40823	53.89279	2.32777
6	Gaussian	70924.73115	1.35813	49059.64927	55.64808	3.93228
7	Gaussian	6268.55869	0.60313	9763.99779	57.73329	0.34755
8	Gaussian	49457.73339	1.22195	38023.28101	60.74307	2.74208
9	Gaussian	21565.44385	1.79763	11270.03724	63.133	1.19565
10	Gaussian	24013.28922	1.24876	18065.0732	65.65417	1.33137
11	Gaussian	53346.53285	1.58698	31579.32125	69.56888	2.95769
12	Gaussian	148931.37886	4.101	34116.49124	72.7186	8.25719
13	Gaussian	38028.82656	3.03443	11773.45987	76.78778	2.10843
14	Gaussian	24161.40656	1.04996	21618.15811	80.97423	1.33958
15	Gaussian	71391.55946	3.79839	17656.92805	83.75877	3.95816
16	Gaussian	17591.18955	1.93111	8557.6814	88.74311	0.97531
17	Gaussian	238836.8357	3.78442	59288.33322	94.22666	13.24182
18	Gaussian	96055.6852	3.04124	29671.56252	104.36562	5.32561
19	Gaussian	29964.93466	4.82372	5835.77481	113.30485	1.66134
20	Gaussian	54494.05535	1.96741	26020.83444	119.21882	3.02131
21	Gaussian	23293.63334	2.8256	7789.04128	123.82091	1.29147

Figure 1. Resolution power analysis of a representative separation profile from a single-cell lysate. a Peak fitting of the single-cell proteome profile across 35–128 kDa range. The proteome profile (black line) is overlaid with the fitted model (red line), composed of 21 Gaussian peaks (green curves). Peaks were identified using second derivative-based detection following baseline subtraction. **b** Table summarizing the fitted parameters for each peak, including peak area, position, height, and full width at half maximum (FWHM). Peak fitting was performed using constrained nonlinear regression with the following bounds: (1) peak position ±5%, (2) FWHM ±50%, and (3) peak height ±50% of the initial estimates generated by OriginPro.

2. Even the resolved peaks are probably multiplets. Thus, the difference between two proteomes cannot be attributed to a difference in abundance of one or even several proteins; massive shifts in protein

abundance result in a small variation of the overall envelope. Even when the authors can see a shift at a certain MW, they are uncertain what protein this can be attributed to. An example they provide on p. 10 where they wildly speculate, but unable to tell for sure, the protein identity, is a case in point.

We thank the reviewer for the comment. We agree that peak multiplicity and the lack of direct protein identification are inherent limitations of single-cell PAGE-PISA. The primary goal of our method is to sensitively capture electrophoretic band patterns that reflect the overall protein composition and abundance within individual cells, which can be mainly used for unsupervised clustering and classification of cell states. These points have been clarified in the Introduction (lines 84–93, page 4) of the revised manuscript.

3. In contrast, mass spectrometry based single cell proteomics (MS-SCP) can identify and quantify robustly 2000 proteins and with less certainty – up to 5,000 proteins per cell. There is no way that PAGE-PISA can approach these numbers even theoretically.

We thank the reviewer for the comment. We acknowledge that single-cell PAGE-PISA does not currently achieve the same level of proteome identification as MS-SCP. While it is not designed for broad protein identification, we note that single-cell PAGE-PISA provides sensitive and quantitative protein band profiles that are useful for distinguishing and classifying single-cell states (lines 84–93, page 4). This capability may be particularly useful for detecting low-abundance proteins and for resolving specific proteoforms that are accessible through fluorescent labelling techniques (lines 355–368, page 14).

Still, as said, I liked this paper, because:

1. It's a daring enterprise where, using commercial parts (e.g., 8 cm PAGE) clearly not designed to handle such small sample volumes, one achieves spectacular results and proves beyond reasonable doubt that these results are real.

We thank the reviewer for the positive comment and for recognizing our efforts to push the limits of polyacrylamide gel electrophoresis for single-cell proteome profiling analysis.

2. The authors provide a spectacular range of ideas on how to improve their technology. Even though I don't think any of this will help PAGE-PISA to become competitive with MS-SCP, it demonstrates their ingenuity and creativity.

We are grateful for the reviewer's recognition of our efforts to explore new possibilities in advancing single-cell proteome quantification.

What I didn't like, however, is the overselling PAGE-PISA. The authors should be more realistic and provide data on the peak capacity of their "spectra" as well as the mass resolution. They should also acknowledge that PAGE-PISA is no match to MS-SCP and unlikely to be ever.

We thank the reviewer for the comment. In response, we have revised the manuscript according to the points addressed in our previous responses. The argument on peak capacity and mass resolution has been added to Supplementary Fig. 6a and 6b and is described in lines 205–210 on page 8. Furthermore, the limitations of our method regarding protein identification have been clarified in the revised manuscript (lines 392–400, page 15–16). A comparison with MS-SCP is also included in Supplementary Table 1.

I was also put down by the fact that on Figure 2f the UMAP analysis separated two cell lines only in the 3d, least significant, dimension. Even though I am not surprised, for the authors it should have been a wake-up call demonstrating that their technique produces barely usable data

We thank the reviewer for raising this important point regarding the UMAP analysis. We acknowledge that the original 3D UMAP plot (Figure 2f in the main manuscript) may have given the impression that the separation between PC-3 and U2OS cell lines occurs only along the third, least significant dimension. To clarify this, we have re-examined the UMAP results and confirmed that the two cell types also show clear separation in 2D projections, specifically in UMAP 1 vs. UMAP 2 and UMAP 1 vs. UMAP 3 plots, indicating that the clustering is not confined to higher-order dimensions. These 2D projections are now included in the revised Figure 2 (panels a and b), and we have replaced the original 3D UMAP plot with the 2D version in Figure 2f to improve interpretability. We believe these revisions more accurately reflect the biological differences captured by our method and address the reviewer's concern. The relevant discussion has been updated in lines 221–225, page 9.

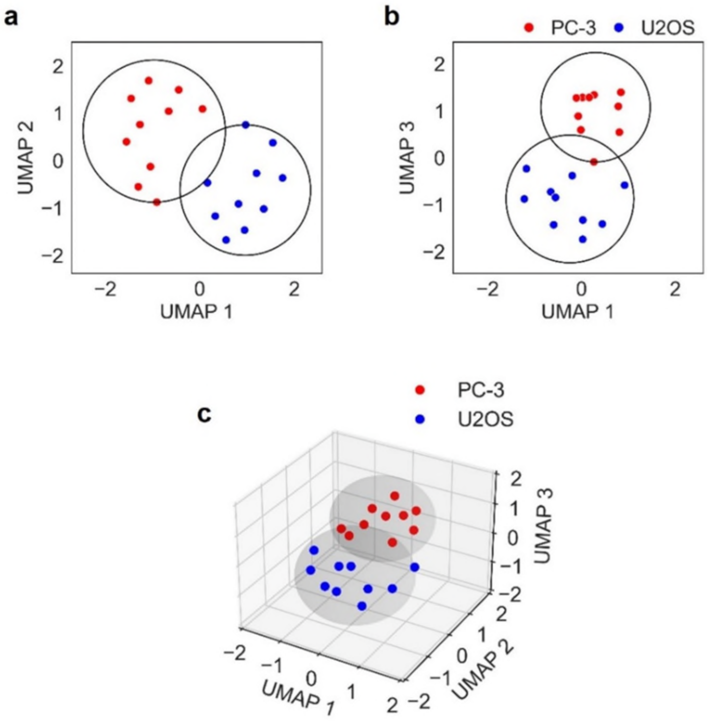

Figure 2. UMAP projection of single-cell proteomic profiles. a, b Two-dimensional UMAP projections based on electrophoretic protein band profiles from individual U2OS (blue) and PC-3 (red) cells, shown as UMAP 1 vs. UMAP 2 (**a**) and UMAP 1 vs. UMAP 3 (**b**). **c** Three-dimensional UMAP projection. Clusters were identified using *k*-means clustering. Number of biological replicates: n = 10 for U2OS and n = 10 for PC-3.

But as said, if the authors provide a more sober account of their technique, they will deliver an interesting read and an inspiration for great many scientists.

We thank the reviewer for the constructive feedback and encouraging remarks on our manuscript. We hope the revised version presents a balanced and realistic perspective on the technical strengths and limitations of single-cell PAGE-PISA as an alternative approach for single-cell proteome profiling.

Our response to Reviewer #2:

We sincerely thank the reviewer for raising many critical points regarding our development. As outlined in the following responses, we have carefully considered all comments in our revision. The reviewer's remarks are presented in ***bold italics***, followed by our responses. Modifications in the revised manuscript are highlighted in red font.

Major points:

1. Throughout the paper, the term "single-cell proteomics" is used in a misleading way. What the authors here call "single cell proteomics" is rather the profiling of the major bands after PAGE for what appear to be single cells. The authors should find a more appropriate and less misleading term since there are MS-based single-cell proteomics technologies that provide protein identities for several hundred species and relative levels for several of them in single isolated cells.

We thank the reviewer for raising an important point regarding the use of the term *"single-cell proteomics"*. To avoid confusion with identification-based approaches such as MS-based single-cell proteomics, we have revised the manuscript to use the term *"single-cell proteome profiling"*. This term more accurately reflects the molecular weight-based, gel-resolved profiling enabled by single-cell PAGE-PISA, which provides quantitative separation patterns across the entire proteome of individual cells. While our method does not provide protein identities, it enables resolution of proteoform-level differences and supports downstream analyses such as clustering, differential expression, and trajectory inference. We have added an explanation of this revised terminology in the Introduction (lines 84–93, page 4) to clarify the scope and capabilities of our approach.

2. The authors acknowledge that MS-based single-cell proteomics technologies exist and that they are capable of detecting 10^4 copies per cell. One aspect that they misleadingly present is that these technologies also provide protein identity information, which is crucial for comparing the two methods. This calls into question the usefulness of their method, which provides a "fingerprint" for each cell, but not protein identity information.

We thank the reviewer for highlighting this important point. In response, we have revised the opening paragraph of the manuscript to clearly focus on single-cell proteome profiling rather than protein identification (lines 40–49, page 2). The revised text now distinguishes between identification-based

approaches, such as MS-based single-cell proteomics, and quantification-based approaches, such as ours, which aim to profile the abundance of a broad range of proteins in single cells to support clustering and pseudotime trajectory analysis (lines 84–93, page 4).

We also understand the reviewer's concern regarding the lack of direct protein identification and its implications for the overall usefulness of the method. While this is a limitation of single-cell PAGE-PISA, we believe the method remains valuable in contexts where molecular identity is not essential. It provides highly sensitive and reproducible electrophoretic band patterns that reflect global protein abundance and molecular weight distribution. These quantitative profiles enable biologically meaningful comparisons between single cells, allowing for clustering, trajectory inference, and identification of functional cell states solely based on proteome-level variation (lines 84–93, page 4).

Looking ahead, we believe the method has potential to further enhance its molecular interpretability. For example, selective labelling of a small number of proteins may allow unambiguous identification of specific bands, similar in concept to a protein ladder (lines 392–400, page 15–16). Fluorescent probes targeting specific post-translational modifications could also enable proteoform-level resolution without enrichment. Furthermore, applying native PAGE conditions allows for the analysis of intact protein complexes, preserving non-covalent interactions and enabling structural aspects of the proteome to be assessed (lines 355–368, page 14). These directions may expand the applicability of single-cell PAGE-PISA to more specialized single-cell proteomic questions, while maintaining its strengths in simplicity, sensitivity, and quantitative robustness.

3. Related to the previous point, although the method could be used to distinguish different cells in a population, the approach seems extremely laborious for this purpose. In other words, simple staining for a few cell markers would provide this type of information (either by microscopy or FACS). The authors could claim that "fingerprinting" the proteome would provide a more reliable way to cluster different cell types, but no application is actually provided, making the utility of this method rather difficult to understand. Can the authors speculate on the practical usefulness of their method?

We thank the reviewer for the comment. We agree that, compared to fluorescence imaging or antibody-based staining, single-cell PAGE-PISA may appear more labour-intensive for distinguishing cell types. Nonetheless, our approach enables comprehensive proteomic fingerprinting at the single-cell level that extends beyond basic clustering based on a few known markers.

For instance, in our study of iPSC-derived cardiomyocyte differentiation, single cell PAGE-PISA enabled us to observe gradual proteomic changes across single cells that aligned with the progression from pluripotency to differentiated states (Figures 3e and 3f). By combining these quantitative profiles with

pseudotime analysis, we could resolve transitional subpopulations along the differentiation trajectory that may not be fully resolved using a limited set of known markers. This level of temporal and molecular resolution is particularly useful when studying dynamic biological processes where canonical markers are insufficient to capture intermediate states.

Beyond this system, we consider potential applications in immune cell analysis, such as profiling of T-cell heterogeneity. T-cell exhibits diverse subpopulation states, many of which lack clear markers for isolation (P. Savas et al., *Nat. Med.* **24**, 986 (2018); N. Slavov, *Curr. Opin. Chem. Biol.* **60**, 1 (2021)). Thus, by profiling the full proteomic content of each cell, single-cell PAGE-PISA can help detect rare or intermediate states defined by functional, not just phenotypic differences.

Furthermore, single-cell PAGE-PISA may complement existing cell marker-based approaches by uncovering features such as novel biomarkers, previously unknown native protein complexes, and low-abundance phosphorylated proteins. These strengths position single-cell PAGE-PISA as a valuable tool with broad applications in both biological and clinical research. We have added this discussion in lines 355–368, page 14.

4. At the current stage we obviously see the importance of the technological development of the PISA method (https://doi.org/10.1101/2024.12.05.625331) as an interesting technical application, but the current paper should stand alone. Although it has a lot of potential and we think a little more work could make it very interesting, at this stage we are struggling to understand its power in the context of cell phenotyping. One way to demonstrate the use of the method is that in direct comparison with MS-based single cell proteomics, it has some advantages (throughput? Real sensitivity in both identification and measurement of low abundance proteins? etc.). This is not the case in its current form. For example, there is no indication of protein identities and no indication that the method can actually identify (with their molecular identity) low abundance proteins as suggested. Can the authors measure the identity of different bands from the bulk and use this as a possible way to interpret at least some phenotypic changes in the single cell environment? We would find a similar application potentially interesting and at least could be more directly compared and benchmarked with MS-based single cell proteomics technologies, possibly providing a reason why this method would be preferable.

We thank the reviewer for the thoughtful comment. We fully agree that for this method to be broadly useful in cell phenotyping, it is essential to demonstrate clear advantages over MS-based single-cell proteomics. We also acknowledge that, although our optical system enables single-molecule sensitivity, the

ability to resolve distinct protein peaks is currently limited by the resolution of electrophoretic separation rather than by the optical detection capability itself.

Although discussed in earlier responses, we would like to reiterate several key distinctions from MS-based approaches. Single-cell PAGE-PISA enables absolute molecular quantification by directly counting fluorescently labelled molecules and offers flexibility in labelling strategies. In contrast to peptide-based workflows in MS, our system allows for selective profiling of post-translationally modified proteins (e.g., phosphorylated isoforms using Phos-tag gels) and detection of native protein complexes under non-denaturing conditions. These capabilities provide structural and modification-specific insights that complement MS-derived proteomic data (lines 355–368, page 14). To facilitate comparison, we now include a summary table (Table 1) outlining key differences in quantification principle, throughput, and detection sensitivity between single-cell PAGE-PISA and MS-based single-cell proteomics.

Regarding protein identification, we recognize this as a current limitation of single-cell PAGE-PISA. As detailed in response to Reviewer #1, peak fitting of a representative single-cell proteome profile revealed 21 Gaussian peaks between 35 and 128 kDa (Figure 1b; Supplementary Fig. 6a and 6b; lines 205–210, page 8), corresponding to up to 21 resolvable protein bands under current conditions. As also described in response to comment #9, each peak represents proteins with a copy number of at least 10^5–10^6 per band, which reflects the current resolution-limited detection threshold. Although NHS-based labelling tends to favour high-abundance proteins, we anticipate that selective labelling strategies such as the use of multiple antibodies or reactive groups targeting specific functional classes could reduce band overlap and allow for the detection of lower-abundance proteins with a similar number of identifiable targets as with NHS labelling (lines 394–396, page 15).

Further development may expand this range. Improved separation (e.g., two-dimensional PAGE) and multiplexed labelling with spectrally distinct fluorophores may enable identification of a greater number of proteins per cell. Additionally, although current single-cell PAGE-PISA imaging with Cy5 samples detect ~1% of the total proteome, we confirmed that this fraction can be increased to >10% using brighter fluorophores such as SeTau-647 (K. Podgorski et al., *PLoS One* **7**, e51980 (2012); Figure 3), thereby expanding the dynamic range and improving sensitivity for low-abundance proteins often missed by MS due to ion suppression or sample loss during enrichment. We have added these points in the revised manuscript (Supplementary Fig. 10, lines 349–354, page 14).

In response to the reviewer's suggestion, we believe that molecular identification of specific bands is indeed possible using bulk cell lysates. For example, candidate bands observed in single-cell PAGE-PISA profiles can be matched to bulk MS data by first labelling intact cells with antibodies against candidate proteins, enriching the labelled population using FACS, and then identifying the proteins via MS in pooled samples. This integrative approach allows phenotypically meaningful bands to be molecularly annotated

and can aid in interpreting cell state differences observed in single-cell PAGE-PISA. We have added this point to the revised manuscript in lines 392–400, page 15–16.

In summary, while single-cell PAGE-PISA does not currently match the proteome depth or identification capabilities of MS-based methods, it offers complementary strengths such as absolute quantification, tunable labelling selectivity, and compatibility with phenotype-guided workflows. These features make single-cell PAGE-PISA a valuable addition to the single-cell proteomics toolkit, especially for applications requiring proteoform-level, structural, or spatial information.

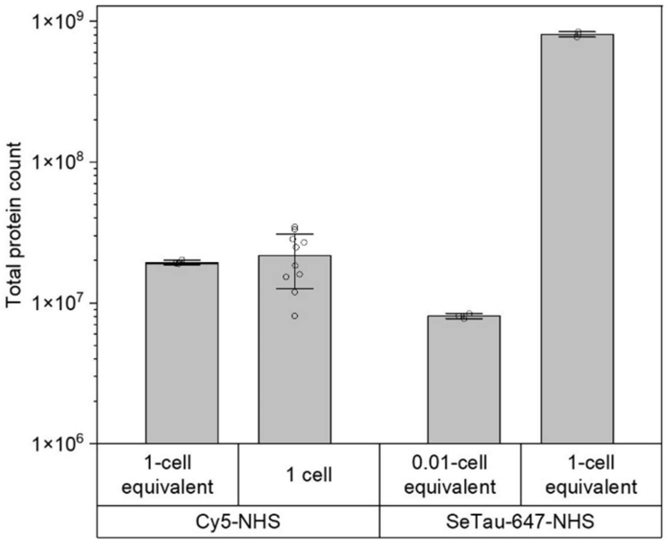

Figure 3. Comparison of total protein count using Cy5 and SeTau fluorescent dyes. Cy5-NHS ester dye was used to label either single-cell lysates (from individual cells) or bulk cell lysates diluted to 1-cell equivalent. SeTau-647-NHS ester dye was used to label bulk cell lysates at 1-cell or 0.01-cell equivalent. SeTau-labelled 1-cell equivalent cell lysates showed a 1–2 order of magnitude increase in total protein counts compared to Cy5-labelled cell lysates. Error bars represent ± standard deviation. Number of biological replicates (single cells): n = 10, number of technical replicates (bulk cell lysates): n = 3.

Table 1. Comparison between single-cell PAGE-PISA and MS-based single-cell proteomics approaches

Feature	Single-cell PAGE-PISA	MS-based single-cell proteomics
Quantification	Provides absolute molecular counts via single-molecule fluorescence imaging. Quantifies approx. 1% of the total protein abundance.	Provides relative abundance via label-free or isotopic labelling.
Sensitivity	Detects 10^4–10^5 copies per protein species.	Detects 10^4–10^5 copies per protein species.
Protein identification	Currently, 20–60 protein bands can be resolved simultaneously via molecular weight. No direct protein identification but possible with antibody-based detection directly on gel or in combination with FACS and conventional MS.	Identifies >3, 000 protein groups via mass spectra and database matching.
Throughput	20 cells per experiment but can be scaled up to ~1,000 cells through automation within 30 hours.	High-throughput processing of 100–1,000 cells within several days.
Cost & workflow	Cost-effective and straightforward ($0.6–1/cell)	Expensive and technically complex ($2–3/cell)
Applications	Whole proteome profiling, analysis of intact, native, modified, and spatially confined proteins	Proteome-wide identification, de novo discovery, spatial proteomics through imaging

5. Phenotypically, the differences during cardiomyocyte differentiation are very large, and fluorescence imaging or simply staining for a few proteins such as MyoD and myosins will provide very similar information for thousands of cells, providing equally reliable staging and grouping of cells. We think the most interesting part of the work would be to identify a subpopulation of cells that are not easily distinguishable, or alternatively, to provide real molecular information about the bands that are identified (which is missing in this work). This is now possible with MS-based single cell proteomics. Can the authors provide an application where this method would outperform MS-based single cell proteomics? We think this would be possible, for example, if it also quantifies aspects that are more difficult to measure with MS-based single-cell proteomics technologies, such as specific protein modifications or protein complexes (in non-denaturing gels) that are not easily seen with these technologies. Overall, we think that the authors should find an obvious application that makes this method useful and allows us to measure or identify something that could not be seen or measured at the single cell level before.

We thank the reviewer for the insightful comment. We agree that the key value of our method lies in identifying subtle subpopulations that are not easily distinguishable by standard markers or in revealing the molecular content of protein bands, particularly in ways that complement or exceed the capabilities of MS-based single-cell proteomics.

As the reviewer has suggested, one such application is the quantification of post-translationally modified proteins. For example, integrating Phos-tag PAGE (E. Kinoshita et al., *Nat. Protoc.* **4**, (2009)) allows us to separate and quantify phosphorylated isoforms of proteins at the single-cell level. This is especially relevant in immune cell studies, such as T cells and macrophages, where activation and functional potential rely on the phosphorylation states of signaling proteins like ZAP-70 and STATs. Such phosphorylation differences often exist between phenotypically similar cells based on surface markers but undetectable in peptide-level MS workflows. Hence, single-cell PAGE-PISA captures these differences as intact proteoforms, enabling more nuanced functional phenotyping.

We also agree that the native protein complexes represent another important and underexplored class of targets. Single-cell PAGE-PISA can preserve non-covalent interactions through native PAGE conditions, enabling analysis of intact complexes in single cells. Such analysis is particularly valuable for detecting signaling assemblies like those formed in tumour necrosis factor (TNF) receptor pathways, where binding of TNFα to TNFR1 or TNFR2 induces formation of distinct protein complexes that drive survival, apoptosis, or necroptosis (P. Gough et al., *Front. Immunol.* **11**, (2020)). In fact, recent findings have demonstrated that intrinsic differences in TNFR1 expression and clustering at the single-cell level contribute to the heterogeneous activation of NF-κB in response to TNF-α (C. T. Cheng et al., *iScience* **27**, (2024)). Thus, by analyzing these complexes in their native state, single-cell PAGE-PISA not only reveals heterogeneity

fquantification of such complexes, which is crucial for uncovering threshold effects, dose-dependent responses, and stoichiometric imbalances that alter cell fate decisions within heterogeneous cell populations.

Furthermore, we note that single-cell PAGE-PISA enables spatially resolved subcellular proteomics through the use of proximity-based labelling strategies. This approach is especially useful for studying organelle-specific proteomes such as that of mitochondria. For instance, photoactivatable probes that accumulate in mitochondria can selectively tag mitochondrial proteins, which can then be analyzed by single-cell PAGE-PISA. Since mitochondrial proteins constitute only ~5% of the total cellular proteome, their detection at the single-cell level typically exceeds the sensitivity of current MS-based methods. By contrast, single-cell PAGE-PISA with single-molecule sensitivity can effectively capture these small, compartment-specific proteomes without the need for cell fractionation or extensive enrichment procedures. We have added the discussion accordingly in lines 355–368, page 14.

Taken together, these examples illustrate how single-cell PAGE-PISA can access biologically meaningful molecular features that remain difficult to resolve by MS-based single-cell proteomics alone.

6. Figure 4 from the PISA method and the description of the method itself (https://doi.org/10.1101/2024.12.05.625331), which are not yet published, are important to provide information about the sensitivity of the method. Similarly, the description of the details of the imaging method is not peer-reviewed per se. The authors may be in the process of publishing the original paper, but if this is not the case, the current paper would be missing an important set of information and validations, so we feel somewhat confused as we see this paper as a continuation of previous work that may have clear limitations in its unpublished form and may never be published.

We thank the reviewer for raising this important point regarding the status of the PISA method and the associated preprint (https://doi.org/10.1101/2024.12.05.625331). While the preprint describes the optical design of the PISA light-sheet microscope and includes initial demonstrations of gel-based imaging, the current study is focused on an entirely separate biological application: single-cell proteome profiling using PAGE separation and single-molecule quantification. Although the same microscope is used, all components of the experimental and analytical workflow presented in this manuscript, including single-cell lysate sample preparation, SDS-PAGE separation, dye labelling, imaging, data processing, clustering, and pseudotime analysis, have been independently developed, optimized, and validated within the present study. Therefore, the current manuscript is self-contained and does not rely on unpublished results from the earlier preprint.

Importantly, although the preprint itself has not been published, its core optical design has been adopted in cutting-edge commercial microscopy systems and has been widely used by the research community since 2020, for example, in the Zeiss Lattice Lightsheet 7 microscope.

In response to the reviewer's comment, we have also included new validation experiments, including spike-in sensitivity and linearity tests, which further support the robustness and reproducibility of the method. We hope this clarifies that the present manuscript can be fully evaluated on its own merits, independent of the publication status of the earlier preprint.

7. One question is about a central point that is also importantly raised by the authors and that remains. Rare subpopulations of cells with distinct properties are often masked at the bulk level, but the throughput proposed by the authors is relatively low, and the authors do not use their method to identify rare populations. What is the current throughput of this method? Would it be possible to scale this up to thousands of cells? If so, at what cost per cell and how long would it take to do so? The authors should discuss at least some of these aspects more openly and possibly refocus the introduction (see minor points).

We thank the reviewer for the comment regarding the overall throughput and scalability of our method. At present, single-cell PAGE-PISA can analyze 20 single cells per experiment that includes manual single-cell isolation (40 minutes), sample preparation (100 minutes), SDS-PAGE (70 minutes), and single-molecule imaging (200 minutes), totaling approximately 7 hours. We believe scaling up to thousands of cells is feasible through automated single-cell collection and sample preparation, along with rapid polyacrylamide gel electrophoresis.

For instance, fluorescence-activated cell sorting (FACS) or an automated live imaging and cell picking system (ALPS) (J. Jin et al., *Proc. Natl Acad. Sci. USA.* **120**, (2023)) can dramatically improve sample throughput by isolating thousands of single cells into a multi-well plate within tens of minutes. To facilitate simultaneous sample preparation for thousands of single cells, robotic dispenser can be utilized to deliver the reagents into the multi-well plate, albeit the time for the protein labelling and removal of unreacted dyes remain the same. In addition, the electrophoresis can be run parallelly using multiple electrophoresis kits and the gel run time can be reduced from 70 minutes to 10 minutes with rapid PAGE (400 V) without compromising the separation resolution. While imaging currently limits the overall throughput, with each single-cell measurement takes about 10 minutes (80 μm/s), our preliminary test demonstrates that scan speeds can be increased up to 400 μm/s without compromising quantitative accuracy. By incorporating the state-of-the-art complementary metal-oxide-semiconductor (CMOS) camera with a 3-fold larger field-of-view than the current set-up, data acquisition speed and coverage during imaging can also be improved.

Thus, scaling up to thousands of cells would take approximately 30 hours, from single-cell isolation to single-molecule imaging.

At present, the cost is estimated to be between $0.60-1.00 per cell but can be reduced to $0.11/cell if commercial gradient gels are to be replaced with homemade gels. We believe that large-scale implementation to thousands of cells will remain cost-effective compared to other single-cell proteomics. Consequently, we have included this discussion in the revised manuscript in lines 379–391, page 15.

8. The reproducibility of running small amounts for bulk lysates that "simulate" single cell concentration is largely biased by the more intense bands (more abundant proteins), but one of the larger claims of the authors is the ability to see and detect very low abundance proteins. The authors should design a "lysate spike-in experiment" to show that spiking even small amounts of an exogenous protein (GFP?) into the complex cellular mixture can indeed provide reliable detection of low abundance proteins as also defined by the authors (10^2-10^3). In doing so, the authors should adjust the total fluorescence so that the band of the exogenous protein is likely to be smaller than the ~100 more abundant proteins, all of which are likely to be more abundant than 10^4. Considering the blurred signal in Fig. 2b, we don't think a protein in this range can be visualized at all, as it would probably be difficult to see it in a normal SDS-PAGE where the signal comes from thousands of cells (similar to Supp. Fig. 2a).

We thank the reviewer for the insightful comment. We agree that direct validation of sensitivity under realistic conditions is essential to support our claim of detecting low-abundance proteins. To address this, we conducted a lysate spike-in experiment using transferrin (77 kDa) as a model protein and evaluated its detectability within a complex cell lysate environment (Figure 4a). After subtraction of the negative control, we successfully detected as little as 5 fg of transferrin, corresponding to approximately 7×10^4 protein molecules (Figure 4b). These results demonstrate that single-cell PAGE-PISA can reliably detect and quantify proteins present at copy numbers ranging from 10^4–10^5 within a complex cell lysate background. With further optimization such as improved electrophoretic resolution or enhanced labelling efficiency, we anticipate that the detection sensitivity could be extended down to 10^2–10^3 molecules per protein species. The experimental details have been added in the Method in lines 441–448, page 17 and data have been included in Supplementary Fig. 3 and are discussed in lines 166–172, page 7 and 352–354, page 14 of the revised manuscript.

a

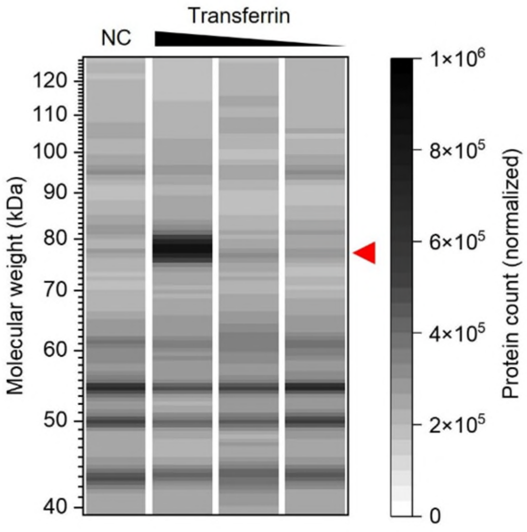

b

Amount of transferrin	Protein counts (theoretical)	Protein counts (detected)
500 fg	3.9×10^6	2.7×10^6
50 fg	3.9×10^5	3.1×10^5
5 fg	3.9×10^4	7.0×10^4

Figure 4. Detection of low-abundance protein by single-cell PAGE-PISA using cell lysate samples spiked with purified transferrin. a Electropherogram of lysate spike-in samples, shown across 39–128 kDa. The lanes represent negative control (NC) and cell lysates spiked with 500 fg, 50 fg, and 5 fg transferrin. Each electropherogram represents the average protein counts across triplicates, normalized to control. The red arrow indicates the transferrin protein band at 77 kDa. **b** Quantitative comparison between theoretical and average protein counts of transferrin detected after subtraction of the negative control. Number of technical replicates: n = 3 per condition.

9. We are confused by Supp. Fig. 3: are diluted bulk also run in parallel with single cells? If not, this should be added to show that the dilution is indeed reliable (experiments in Supp. Fig. 2 are reliable). Similarly, to show the true ability of the method to linearly discriminate the intensity of single cells, an experiment with 1, 2, 4 and 8 cells should be performed.

We thank the reviewer for the comment. We would like to clarify that the diluted bulk cell lysates and single-cell lysates samples were not run on in parallel. However, both datasets were acquired under the same electrophoresis (250 V, 30 mA, and 70 minutes) and microscopic conditions (1,000 mW and 80 µm/s), making them directly comparable. We acknowledge that the previous data showed inconsistencies in protein

counts between diluted bulk and single-cell lysates, particularly in the PC-3 measurements. In response, we have repeated the measurement of bulk PC-3 cell lysates diluted to single-cell level under the same conditions as the single-cell measurements. The updated results now show consistent protein counts and demonstrate the reliability of the dilution-based comparison (Figure 5). We have now included the revised figure in Supplementary Fig. 4c and updated Figure 2e.

To assess the linearity of the method, we performed an experiment using highly diluted bulk cell lysates equivalent to 1, 2, 4, and 8 cells. We used diluted bulk cell lysates instead of actual single cells to minimize the variability from cell-to-cell differences in protein content or cell cycle stages, thus ensuring controlled and consistent protein input. Figure 6a shows the reconstructed electropherogram based on the average raw protein count across three technical replicates. Our result demonstrated excellent linearity (R^2 = 0.998) between cell equivalents and total protein counts (Figure 6b). We have added these data in Supplementary Fig. 2g and 2h and discussed the results in lines 159–165, page 6–7.

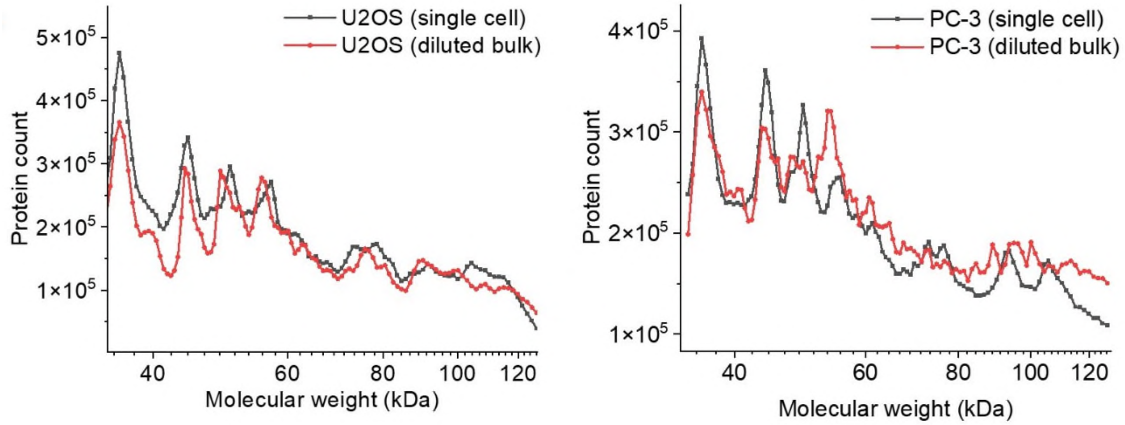

Figure 5. Comparative analysis between single cells and highly diluted bulk cell lysates equivalent to single-cell level. Average proteome profiles across molecular weights for U2OS (left) and PC-3 (right) cells, comparing single-cell lysates (black line) with bulk cell lysates diluted to single-cell level (red line). The proteome profiles were averaged independently across multiple single-cell lysates and across technical replicates of bulk cell lysates diluted to single-cell level, respectively. Number of biological replicates (single cells): n = 10 for U2OS and n = 10 for PC-3, number of technical replicates (bulk cell lysates): n = 3 for U2OS and n = 3 for PC-3 cells.

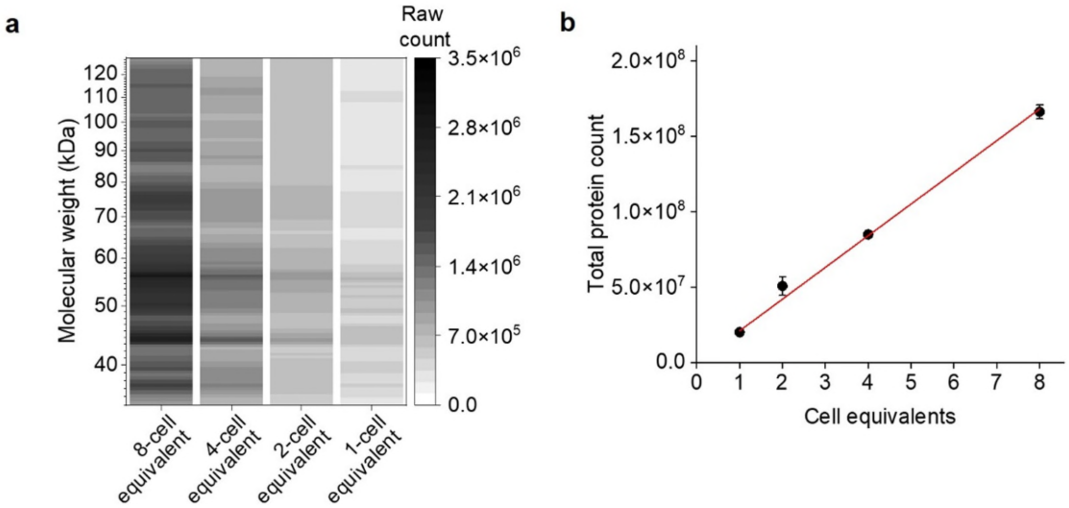

Figure 6. Linearity of single-cell PAGE-PISA quantification using diluted bulk U2OS cell lysates. a Electropherogram of bulk U2OS cell lysates diluted to 1, 2, 4, and 8 cells equivalent, shown across 35–128 kDa. Each electropherogram represents the average raw protein counts across triplicates. **b** Linearity between cell equivalents and total protein count. Error bars represent the standard error of the mean. Number of technical replicates: n = 3 per cell equivalents.

Minor points:

1. The title is misleading. As single-cell proteomics is becoming a reality, such a paper should have a title that more accurately reflects the actual content and clearly states that the main topic is the development of a single-cell PAGE approach for the analysis of protein bands after N-hydroxysuccinimide-ester dye staining.

We thank the reviewer for the comment. We agree that, as single-cell proteomics continues to advance, it is important for the title to clearly reflect the scope and focus of the study. In response, we have revised the title of the manuscript to *"Comprehensive molecular profiling of single-cell proteome via gel electrophoresis and 3D single-molecule imaging"*. The revised title describes the core aspects of our approach, which is based on PAGE separation of proteins labelled via NHS-ester chemistry, followed by single-molecule fluorescence imaging for quantification. We hope this revision addresses the concern and more clearly communicates the nature and contribution of the work.

2. The introduction and especially the first paragraph lack focus and should better reflect the actual strength of the work, which will probably require more careful refocusing based on the actual applications (see main points).

We thank the reviewer for the comment. As suggested, we have revised the introduction to clearly emphasize the relevance of single-cell proteome profiling for the quantification of a wide range of protein abundances in individual cells that enables downstream analyses such as cell state clustering and pseudotime trajectory inference (lines 40–49, page 2 and lines 84–101, page 4).

3. The authors correctly point out that several other groups have developed single-cell Western blots. Likewise, capillary electrophoresis can be used for similar approaches. Can the authors reliably compare their method with modifications of these methods tailored to see the whole proteome (at similar NHS conjugations)? In other words, here the method seems particularly interesting for sensitivity, but "whole proteome visualization" using N-hydroxysuccinimide-ester dye (or other protocols) is not innovative, and several other solutions provide this type of signal. Can the authors provide some sort of comparison with one of these other single cell systems, adapted for the purpose of imaging all proteins, to benchmark their sensitivity? We think this would be helpful to show the superior sensitivity of their method compared to other methods.

We thank the reviewer for the insightful comment. While both single-cell Western blotting and capillary electrophoresis (CE) can be modified to visualize broad proteome features using NHS-ester dyes, they face inherent limitations for single-cell proteome profiling. Single-cell Western blotting, for example, allows high-throughput analysis of over 1,000 cells but suffers from limited separation resolution due to the short migration distance (~1 mm), restricting the number of resolvable bands. In contrast, single-cell PAGE-PISA provides approximately eightfold longer separation, allowing higher-resolution profiling across a broad molecular weight range. Moreover, single-cell Western workflows do not accommodate pre-labeled lysates; intact cells must be gravity-loaded and lysed in situ, making the use of NHS-ester dyes and integration with PISA technically incompatible.

CE systems, such as the PA 800 Plus (Sciex), can in principle provide high-resolution separation because of a longer separation distance. However, current CE workflows are inherently sequential and require 1–2 hours per sample, limiting throughput. Additionally, the low sample volume and sample loss in CE make it difficult to achieve comprehensive proteome coverage at the single-cell level.

Taken together, among NHS-compatible approaches, single-cell PAGE-PISA offers a practical and effective balance of resolution, sensitivity, and throughput for whole-proteome profiling in individual cells. We have added this discussion in lines 369–378, page 14–15.

4. Which statistical test was used in Figure 2d, 3d, and 3i? The authors should mention this in the figure legend.

We thank the reviewer for the comment. The statistical test used in Figures 2d, 3d, and 3i was one-way Welch's ANOVA followed by the Games-Howell post-hoc test. These statistical methods were chosen as it provides robust group-level comparisons for datasets with unequal variances and sample sizes. We have now added this information to the corresponding figure legends.

Response to the reviewers' comments:

<u>Our response to Reviewer #2:</u>

We sincerely thank Reviewer #2 for the thoughtful and constructive comments that have substantially improved our manuscript. We also apologize for not fully addressing several important points in our previous response. To address this, we have conducted additional experiments to strengthen our work. As outlined in the following responses, we have carefully considered all comments in our revision. The reviewer's remarks are presented in bold italics, followed by our responses. Modifications in the revised manuscript are highlighted in red font.

We thank the authors for their diligent and thoughtful revisions. The technical quality of what is performed is clearly visible, and the method is described with the necessary detail.

We thank the reviewers for their positive evaluation of our revisions and work.

Nevertheless, in our opinion, several important issues remain unanswered.

1. The most critical issue is related to the inconsistency between the theoretical potential of the method and its demonstrated utility. In the absence of molecular identification of the fingerprinted proteins, in our opinion the data remains difficult to interpret in biological terms.

We thank the reviewer for this insightful comment. To enable biological interpretation in the absence of direct protein identification, we interpret changes in protein band by integrating single-cell PAGE-PISA data with complementary single-cell omics and immunofluorescence measurements. We first evaluated candidate proteins potentially contributing to the 54.4–58.4 kDa band detected at D30 by cross-referencing our single-cell PAGE-PISA data with publicly available single-cell transcriptomic (C. E. Friedman et al., *Cell Stem Cell* **23**, 586–598 (2018)) and single-cell mass spectrometry datasets (L. Ai et al., *Mol. Cell. Proteomics* **24**, 100910 (2025)). Proteins were filtered based on molecular weight and ranked by abundance in each dataset (top 10 at D30 and D21, respectively). Five candidates were selected for immunofluorescence imaging at D0 and D30 based on these rankings and antibody availability (Table 1). Additionally, we performed positive control staining for cTnT and SOX2 as representative cardiac and pluripotency markers, respectively, as well as a negative control to verify the absence of non-specific secondary antibody binding in fixed samples. The experimental details have been added in the Method in

Table 1. Candidate proteins within 54.4–58.4 kDa in iPSC-CMs identified from transcriptomic and proteomic datasets

Single-cell approach	Reference	Top 10 genes/proteins ranked by abundance
Single-cell RNA-sequencing	C. E. Friedman et al., *Cell Stem Cell* **23**, 586–598 (2018)	**MT-CO1**, PKM, **ATP5B**, **P4HB**, PLD3, PSAP, **PDIA3**, GLTSCR2, UGP2, **PHGDH**
Single-cell mass spectrometry	L. Ai et al., *Mol. Cell. Proteomics* **24**, 100910 (2025)	PKM, **ATP5B**, **PDIA3**, **PHGDH**, **P4HB**, UGP2, VAT1, SPPL2A, CCT8, FSCN1

Proteins selected for immunofluorescence (IF) validation are shown in bold.

lines 529–541, lines 550–555, page 21. The list of top 10 candidate proteins identified from single-cell transcriptomic and mass spectrometry datasets can be found in Supplementary Table 1, whereas the list of antibodies used in immunofluorescence imaging is available in Supplementary Table 3.

Of the shortlisted candidate proteins, MT-CO1 and P4HB showed increased protein abundance at D30 relative to D0, consistent with enhanced mitochondrial respiratory activity and prevention of endoplasmic reticulum (ER) stress and protein misfolding, respectively (D. F. Dai et al., *Stem Cells Int.* **2017**, 5153625 (2017)); I. Jang et al. *eLife* **8**, e44528 (2019)). In contrast, PHGDH showed decreased protein abundance at D30, consistent with reduced PHGDH levels reported in hiPSC-derived cardiomyocytes (T. C. Umei et al., *iScience* **28**, (2025)). PDIA3 abundance also decreased at D30, and given its reported association with proliferative activity (M. Yang et al., *Biomed. Pharmacother.* **173**, 116336 (2024)), this reduction may indicate decreased proliferation in differentiated cardiomyocytes and a transition toward a more post-mitotic state (Figure 1a, b). Overall, these results suggest that the protein band observed within 54.4–58.4 kDa at D30 is predominantly contributed by MT-CO1 and P4HB, whereas PHGDH and PDIA3, despite their high abundance in single-cell transcriptomics and single-cell mass-spectrometry datasets, contribute only minimally to the protein band expression. Furthermore, immunofluorescence imaging also revealed cell-to-cell variability in ATP5B abundance at D30 despite no directional change at the population level, suggesting heterogeneous activation of mitochondrial ATP synthesis at the single-cell level. We have added these data in Supplementary Fig. 9 and discussed the results in lines 277–297, page 11–12.

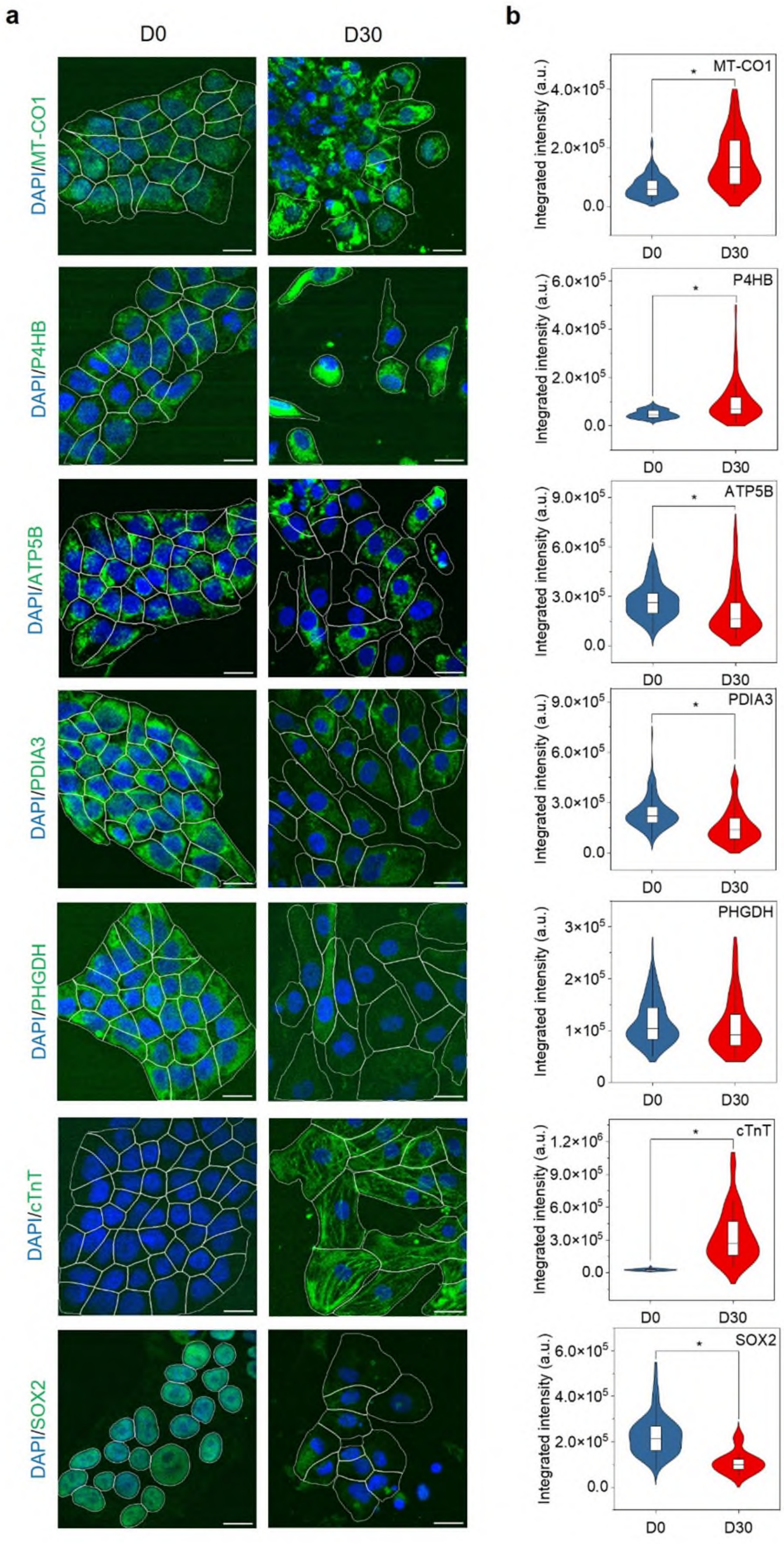
a
D0
D30
DAPI/MT-CO1
DAPI/P4HB
DAPI/ATP5B
DAPI/PDIA3
DAPI/PHGDH
DAPI/cTnT
DAPI/SOX2
b
Integrated intensity (a.u.)
MT-CO1
P4HB
ATP5B
PDIA3
PHGDH
cTnT
SOX2
D0
D30

Figure 1 | Single-cell immunofluorescence analysis of candidate proteins associated with the 54.4–58.4 kDa bands. a Representative immunofluorescence images of cells at D0 and D30 stained for candidate proteins within the 54.4–58.4 kDa range (MT-CO1, P4HB, ATP5B, PDIA3, and PHGDH) along with cTnT (cardiac marker), SOX2 (pluripotency marker), shown in green, with nuclei counterstained with DAPI (blue). Images were acquired as confocal z-stacks (1 µm intervals) and analyzed as sum-intensity projections. Cell boundaries (white outlines) were identified using Cellpose (Stringer et al., 2021); partial and multilayered cells were excluded. Integrated fluorescence intensity per cell was quantified from the green channel. Scale bar: 20 µm. **b** Corresponding violin plots show the distribution of integrated fluorescence intensity per cell (a.u.) at D0 and D30. The box plots show median values (central line), interquartile range (box edges), and whiskers extending up to 1.5 times the interquartile range. Statistical significance was determined by the one-way Welch's ANOVA followed by the Games-Howell post-hoc test. Number of cells per condition were as follows: MT-CO1 (n = 158, D0; n = 26, D30); P4HB (n = 55, D0; n = 63, D30); ATP5B (n = 117, D0; n = 115, D30); PDIA3 (n = 146, D0; n = 87, D30); PHGDH (n = 125, D0; n = 41, D30); cTnT (n = 131, D0; n = 39, D30); SOX2 (n = 84, D0; n = 17, D30). $*p <= 0.05$

While direct molecular identification remains a future goal, we have demonstrated that single-cell PAGE-PISA can provide biologically meaningful insights when integrated with complementary single-cell omics and immunofluorescence data. In this study, protein band changes detected within 54.4–58.4 kDa were cross validated through transcriptomic, proteomic, and immunofluorescence analyses, enabling us to link observed band changes to specific biological processes, namely mitochondrial activity and ER proteostasis during cardiomyocyte differentiation. More broadly, single-cell PAGE-PISA serves as a practical functional screening tool to monitor proteome changes, generate testable hypotheses, and identify molecular weight regions warranting detailed investigation. We have added this discussion in lines 407–416, page 16.

2. The claims of sensitivity (particularly for low-abundance proteins) are not well supported, as clearly in the spike-in experiment in Supplementary Figure 3, where linearity is lost and the middle lane shows no visible signal.

We thank the reviewer for the comment and sincerely apologize for the shortcomings in the data presented in our previous rebuttal. To assess the detection sensitivity of single-cell PAGE-PISA, particularly for low-abundance proteins, we repeated the lysate spike-in experiment using a larger sample size with transferrin (77 kDa) as a model protein and evaluated its detectability in a complex cell lysate background (Figure 2a).

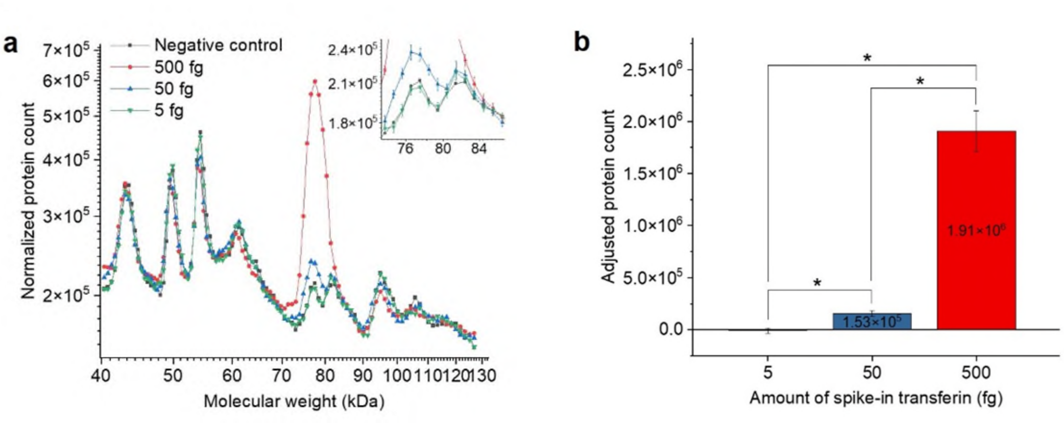

Figure 2. Detection of low-abundance protein by single-cell PAGE-PISA using cell lysate samples spiked with purified transferrin. a Proteome profiles of lysate spike-in samples, shown across 40–128 kDa. The profiles represent negative control (black) and cell lysates spiked with 500 fg (red), 50 fg (blue), and 5 fg transferrin (green). The inset shows a zoom on the main peak of transferrin at 50 fg. Error bars represent ± the standard deviation of the mean. **b** Quantitative analysis shows the total adjusted protein count of transferrin across different spike-in amounts. Statistical significance was determined by the one-way Welch's ANOVA followed by the Games-Howell post-hoc test. Error bars represent ± the standard error of the mean. Number of technical replicates: *n* = 11 for negative control, *n* = 8 for 500 fg, *n* = 9 for 50 fg, and *n* = 8 for 5 fg.

By applying a revised normalization method mentioned below, we now conclude that the system reliably detects transferrin down to 50 fg, corresponding to approximately 1.53×10^5 molecules, although our previous rebuttal stated 5 fg as the detection limit (Figure 2b). These results demonstrate that single-cell PAGE-PISA enables the detection and quantification of proteins present at copy numbers in the 10^5–10^6 range within a complex cell lysate environment. Experimental details are now included in the Methods (lines 463–470, pages 18), with data shown in Supplementary Fig. 3 and discussed in lines 168–174, page 7.

To process the single-cell PAGE-PISA data in a more reliable way, we have substantially revised our normalization approach and applied it consistently across all data in the manuscript. While our initial analysis used *z*-score standardization, we now apply slope-correction normalization to compensate for slope variation between proteome profiles, which likely arises from technical factors such as gel-to-gel variability and minor inconsistencies in gel positioning on the microscope stage. Ideally, the laser excitation path

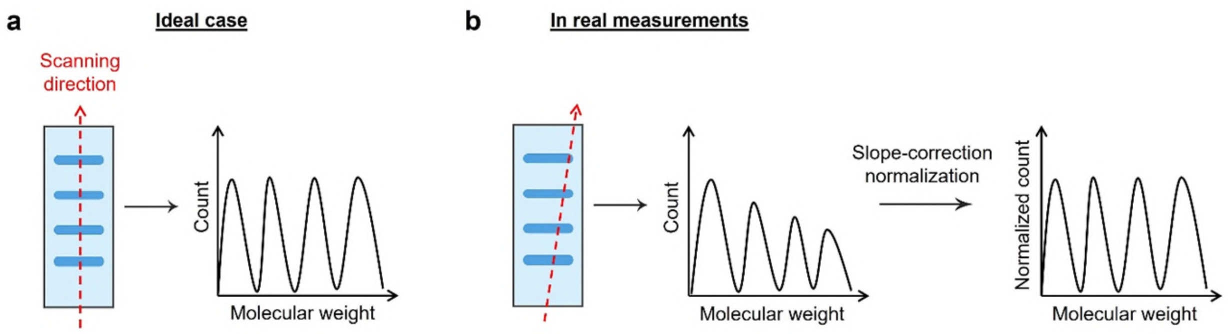

Figure 3. Illustration of how scanning position impact protein quantification. a Ideal scenario where the imaging path (red dashed line) passes through the center of protein bands, yielding a proteome profile in which peaks accurately reflect protein abundance. **b** Imaging path deviates from the band centers causing progressively decreasing protein count that do not reflect actual protein abundance. Slope-correction normalization is required to compensate for this variation.

should pass through the center of each protein band for accurate quantification (Figure 3a). In practice, however, this is inherently difficult for single-cell lysates, as the low protein copy numbers make band positions impossible to determine prior to imaging. Consequently, even small deviations from the band center can introduce slope variations in the reconstructed proteome profiles (Figure 3b).

To correct for slope differences between proteome profiles, we first calculated the ratio of protein counts between a reference (either bulk cell lysates or the average of all single-cell lysates) and individual cell lysates across molecular weights (Figure 4). A linear fit was applied to this ratio to estimate the overall slope, which was then used to correct the protein counts of individual cell lysates. The resulting slope-corrected values are referred to as “normalized protein counts” and are used for comparisons of protein band expressions.

However, slope correction alone removes abundance differences between cells, as all profiles are scaled relative to a reference. To restore protein abundance information after slope correction, we calculated the ratio between the original and normalized protein counts at each molecular weight and applied the mean of these ratios globally to the normalized protein counts. The resulting values, which reflect actual protein abundance with slope bias removed, are referred to as “adjusted protein counts” and are used for comparisons of total protein abundance between cells. The details of the normalization method have been added in the Method in lines 564–573, page 22 of the revised manuscript.

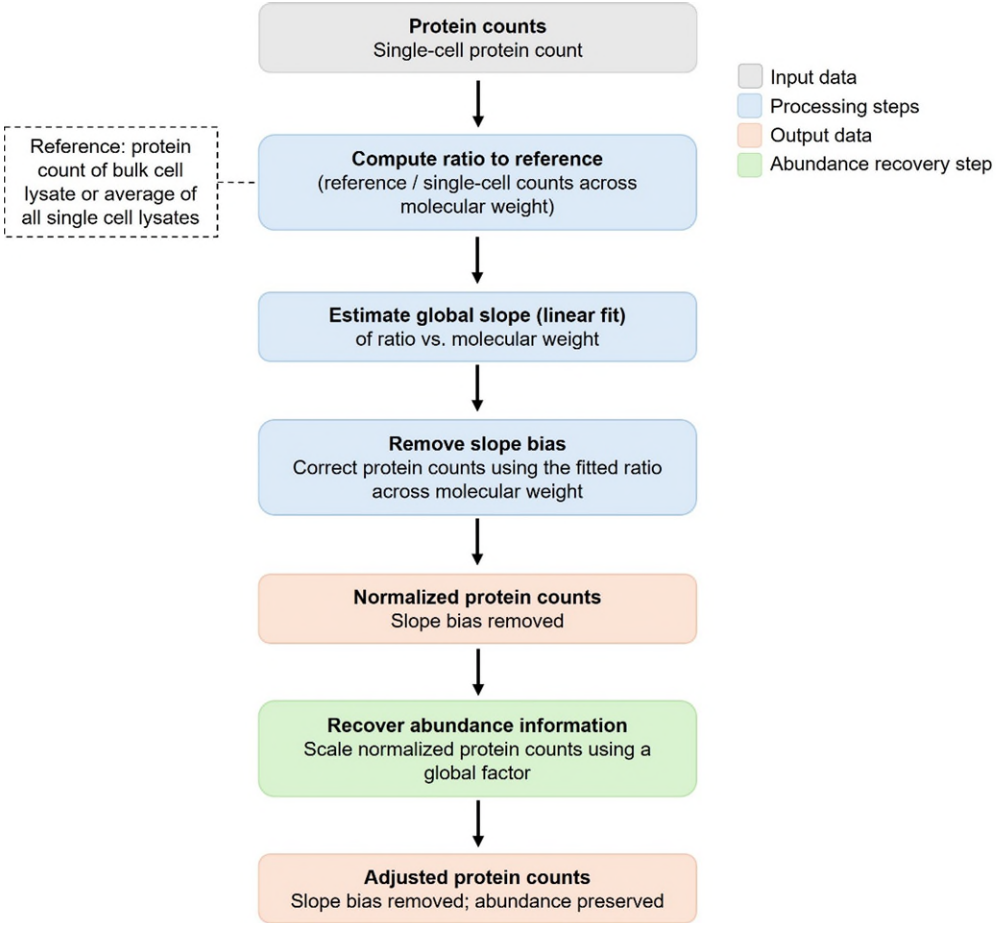

Figure 4. Pipeline of slope-correction normalization in single-cell PAGE-PISA. Protein counts were normalized by calculating the ratio between reference and single-cells across molecular weights, followed by linear fitting to estimate a global slope for profile correction (normalized protein counts). A scaling factor, derived from the mean ratio of original to normalized counts, was then applied globally across molecular weights to restore protein abundance information (adjusted protein counts).

3. In addition, claims of detection of rare populations or minor phenotypes remain to be confirmed without benchmarking and comparison against other established single-cell approaches, including imaging ones.

We thank the reviewer for the insightful comment. Regarding rare populations, we would like to clarify that the current study cannot definitively detect rare populations, as the small number of cells analyzed (typically $n < 100$) precludes adequate sampling of low-frequency subpopulations. Nonetheless, single-cell PAGE-

PISA effectively resolves biologically meaningful subpopulations, as reflected by the clear separation of precursor stage, early, and late cardiomyocyte clusters (Figure 3f in the manuscript). Importantly, these subpopulations are also observed in the single-cell transcriptome data, providing independent confirmation that the subpopulations detected by single-cell PAGE-PISA reflect genuine cellular states (Figure 4a in the main manuscript) (lines 365–374, page 14–15).

Regarding minor phenotypes, we have directly benchmarked our findings against immunofluorescence imaging. Specifically, minor phenotypes detected by single-cell PAGE-PISA in D30 cells showing increased protein band within 54.4–58.4 kDa were independently confirmed by immunofluorescence at the single-cell level (Figure 1a; see also response to Comment 1 for details). The broadened distributions in the D30 violin plots further reflect cell-to-cell heterogeneity, indicating the coexistence of distinct cellular states rather than a homogeneous population (Figure 1b). The directional concordance between single-cell PAGE-PISA and immunofluorescence across multiple protein targets confirms that the minor phenotypes reported in this study are reproducible and biologically meaningful (lines 277–297, page 11–12).

That being said, we do recognize that the research is well done technically and that its novelty and interest for the community are editorial judgments. We leave that to the discretion of the editor in this case and agree with their final decision.

We want to thank the authors for their efforts and hope these comments are useful as they continue to refine and develop their method. We look forward to a future improvement to highlight the strength of this approach for aspects that cannot be solved with other methods.

Response to the reviewer’s comments:

Our response to Reviewer #2:

We sincerely thank Reviewer #2 for the thoughtful and constructive comments that have significantly improved our manuscript. The reviewer’s remarks are presented in bold italics, followed by our response.

The authors have made a substantial effort to address the reviewer’s comments, and the revised manuscript reflects a clear improvement in both technical rigor and clarity. The additional experiments, particularly the immunofluorescence validation and the revised spike-in analysis, strengthen the methodological framework and provide greater confidence in the robustness of the approach.

The authors now offer a more transparent and realistic assessment of the method’s capabilities. The integration of single-cell PAGE-PISA with complementary datasets (single-cell transcriptomics, proteomics, and imaging) represents a thoughtful strategy to enhance biological interpretability in the absence of direct protein identification. While this remains an indirect approach, it is applied carefully and yields biologically coherent insights, particularly in the context of cardiomyocyte differentiation.

The revised sensitivity analysis and normalization procedure are technically sound. Although the detection limits and throughput do not yet match those of cutting-edge single-cell proteomics platforms, the method is now presented in a more balanced and credible way. Similarly, the authors appropriately moderate their claims regarding rare population detection and provide orthogonal validation supporting the observed cellular heterogeneity.

Overall, the work is technically solid.

We thank the reviewer for the positive evaluation of our revised manuscript and for acknowledging the improvements made.
